# The ELBA Force Field for Coarse-Grain Modeling of Lipid Membranes

**DOI:** 10.1371/journal.pone.0028637

**Published:** 2011-12-16

**Authors:** Mario Orsi, Jonathan W. Essex

**Affiliations:** School of Chemistry, University of Southampton, Southampton, United Kingdom; King′s College London, United Kingdom

## Abstract

A new coarse-grain model for molecular dynamics simulation of lipid membranes is presented. Following a simple and conventional approach, lipid molecules are modeled by spherical sites, each representing a group of several atoms. In contrast to common coarse-grain methods, two original (interdependent) features are here adopted. First, the main electrostatics are modeled explicitly by charges and dipoles, which interact realistically through a relative dielectric constant of unity (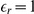). Second, water molecules are represented individually through a new parametrization of the simple Stockmayer potential for polar fluids; each water molecule is therefore described by a single spherical site embedded with a point dipole. The force field is shown to accurately reproduce the main physical properties of single-species phospholipid bilayers comprising dioleoylphosphatidylcholine (DOPC) and dioleoylphosphatidylethanolamine (DOPE) in the liquid crystal phase, as well as distearoylphosphatidylcholine (DSPC) in the liquid crystal and gel phases. Insights are presented into fundamental properties and phenomena that can be difficult or impossible to study with alternative computational or experimental methods. For example, we investigate the internal pressure distribution, dipole potential, lipid diffusion, and spontaneous self-assembly. Simulations lasting up to 1.5 microseconds were conducted for systems of different sizes (128, 512 and 1058 lipids); this also allowed us to identify size-dependent artifacts that are expected to affect membrane simulations in general. Future extensions and applications are discussed, particularly in relation to the methodology's inherent multiscale capabilities.

## Introduction

Over the past decade, coarse-grain (CG) modeling has become an increasingly popular approach to the simulation of membrane systems [1]–[4].

In fact, in recent years, we have developed our own CG model, characterized by two interdependent features which are normally absent from alternative CG force fields [5], [6]. First, the fundamental lipid electrostatics were captured using charges and dipoles, interacting realistically with each other through a relative dielectric constant of unity (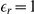). Second, we described water molecules individually using the soft sticky dipole (SSD) potential [7]. These characteristics proved advantageous in a number of important areas. For example, we were able to model the dipole potential, an extremely important property [8]–[12] which is very problematic to measure experimentally [13] and to simulate by alternative CG approaches [14]. Moreover, our model could be coupled straightforwardly with a standard atomistic force field in a multiscale (“dual-resolution”) fashion, allowing the atomistically-detailed treatment of selected parts of the simulation while retaining the CG speed advantage for the surrounding environment [15], [16]. Full details of our original methodology, and specific comparisons with alternative approaches, can be found in recent publications [6], [17], [18].

Despite the encouraging results obtained, our original model also showed a number of limitations. In particular, we recently identified issues related to the Gay-Berne potential [19]–[21], which was used to model the lipid tail sites as ellipsoids [5], [6], [22]. Preliminary studies, aimed at simulating lipid bilayers in the solid (gel) phase, showed the formation of highly interdigitated structures bearing no resemblance to the desired phase (unpublished data). Gel-phase lipids play important roles in many structures (such as the skin) and phenomena (such as microdomain formation), and hence they should ideally be present in a lipid force field. More generally, the Gay-Berne model has a rather complex analytical form, and comprises six independent parameters (for comparison, the popular Lennard-Jones potential is defined by only two parameters); as in any model, it is important to question the need for elaborate components when simpler alternatives are available. We therefore decided to substitute the Gay-Berne representation with the simpler conventional Lennard-Jones potential, which is used to model lipid tails in most alternative CG models [1]–[4]; as shown in this paper, this new representation is indeed capable of reproducing realistic gel phases, while retaining excellent performances in the (biologically prevalent) liquid-crystalline state. Another issue in our original model was that, to capture the hydrophobic effect, the strength of the Lennard-Jones dispersion energy between hydrophilic (water and headgroup) and hydrophobic (tail) sites had to be scaled down with respect to the values determined through the Lorentz-Berthelot (LB) formulae [23]. The LB “combination rules” are simple and physically intuitive, and hence advantageous; in fact, they are commonly used in atomistic force fields [24], [25]. Unfortunately (and in common with other CG models), without ad hoc modifications to the LB rules, preassembled bilayers simulated with our original model were unstable, and dispersions of lipids and water would not self-assemble into membrane structures. The need to resort to such alterations of the general mixing rules was rather unexpected, as one would hope that an accurate water model (such as the SSD [7]), together with an explicit description of the lipid electrostatics, would prove sufficient to capture the hydrophobic effect. Interestingly, it now appears that this issue is associated with the Gay-Berne tails, in relation to their interaction with the Lennard-Jones potentials of headgroups and water. In fact, we show in this work that for the new model (where the Gay-Berne components have been replaced by Lennard-Jones terms), the stability and self-assembly of membranes is achieved without the need to modify the LB rules as described above; the hydrophobic effect no longer needs to be enforced through arbitrary deviations from the LB formulae, but is now an inherent property of the model. While common in atomistic force fields, this notable characteristic of our new model is unique amongst the available CG techniques. As a further improvement of our methodology, in this work we have also considered a simpler alternative to the SSD potential [7] (which we adopted to describe water in our original model [5], [6]). The SSD force field features an octopolar “sticky” term used to reproduce the hydrogen bonding directional properties of bulk water; this (rather complex) potential was partially redundant in our CG force field, because it was only used for water-water interactions. A simpler model, which still includes the essential electrostatics, is the general Stockmayer potential for polar fluids, where a particle is represented by a Lennard-Jones soft sphere embedded with a point dipole [26]. In fact, the Stockmayer model is analytically equivalent to the SSD model when the octopolar “sticky” term is removed. While the Stockmayer potential has been traditionally used to study idealized systems [27]–[30], there have also been two previous applications to specifically model different aspects of “real” water [31], [32]. Here we present a new parametrization of the Stockmayer model aimed at the simulation of liquid water in the context of our CG methodology.

Our new CG force field has been named “ELBA”, an acronym for “electrostatics-based”; this highlights the most important and distinguishing feature of the model, which is its explicit incorporation of charges and dipoles to describe realistically the main electrostatics of the systems. In this paper, we first define the various components of the ELBA force field, and we report on its parametrization. We then test the model's ability to reproduce various experimental observables for a number of different membrane systems, including a gel phase. In particular, we study several physical parameters and processes that are difficult to access by experiment; for example, we investigate the distributions of pressure and electrostatic potential inside the membrane, and we simulate self-aggregation processes. Finally, we summarize the main findings, assess the methodology's limitations, and discuss future prospects.

## Methods

### Water model

**Figure 1:**
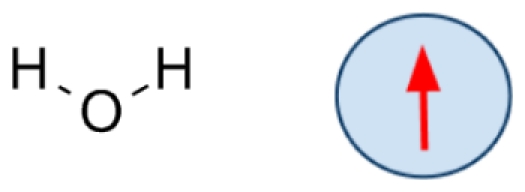
Water coarse-graining. The sketch on the left represents the chemical structure of a water molecule. The corresponding ELBA model is depicted on the right; the arrow represents an electrical point dipole.

The ELBA water model is based on the general Stockmayer potential for polar fluids [26], where a particle is represented by a Lennard-Jones soft sphere (providing excluded volume) embedded with a point dipole (to capture the electrostatics). Each CG water site represents a single water molecule (Figure 1). The total potential energy 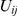 of an interacting pair 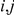 can be expressed as:
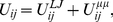
(1)with 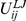 the Lennard-Jones term and 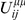 the dipole-dipole term. Both interactions are “truncated”, that is, they are set to zero for interparticle distances larger than a cutoff radius 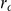 [23]. In molecular dynamics, a well-known problem arising from truncating the interactions is the introduction of a discontinuity in the potential and its derivative (the force); this can affect the energy of the system and induce artifacts in the motion of the particles. This issue can be tackled by altering the form of the potential so that both the potential energy and its derivative go to zero at the cutoff distance [23], [33]. We have therefore adopted a “shifted-force” form of the Lennard-Jones potential [34]:
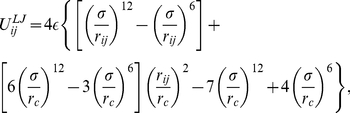
(2)where 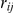 is the interparticle distance, 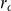 is the cutoff radius, and 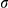 and 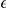 have the standard meaning [23]. For dipole-dipole interactions, we use the classical electrostatic model [23], [35] combined with a cubic “switching” function 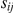 acting between a switching radius 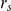 and the cutoff radius 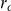 [7]:
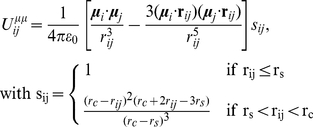
(3)where 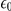 is the electric permittivity of vacuum, 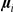 and 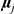 are the dipole moment vectors of sites 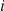 and 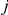, 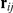 is the distance vector between sites 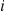 and 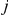, and 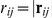.

To simulate the model by molecular dynamics, forces and torques were obtained by differentiating the potentials; explicit expressions are reported in the Supporting Information S1.

### Parametrization and validation

The ELBA water force field comprises six independent parameters: 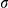, 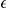, 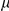, 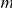, 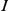, 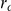. The parameters 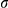 and 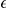 characterize the Lennard-Jones pair interactions (equation 2). The electrostatic parameter 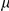 represents the (fixed) magnitude of the dipole moment, which is the same for every water site; referring to equation 3, we can write 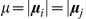. The mass of a water particle is expressed by 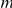, whereas 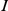 represents the principal moment of inertia (required for the integration of the rotational motion of the dipoles). The parameter 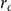 is the cutoff radius, which we assume equal for both the Lennard-Jones and the dipole-dipole potentials. The dipole-dipole switching radius 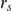 (equation 3) is set to 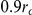, as is conventional [24].

The model was parametrized specifically for bulk water in the liquid phase. The dipole moment magnitude 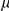 was set to 2.3 D, within the range 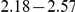 D calculated for the most widely-used atomistic water models [36]–[40]. The cutoff radius 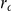 was set to 0.9 nm, for both the Lennard-Jones and the dipole-dipole interactions. The Lennard-Jones parameters and the inertial features (mass and moment of inertia) were obtained through incremental refinements based on the results of trial simulations (this strategy was adopted to parametrize our earlier model [5], [6]). Bulk water systems were simulated at a constant temperature of 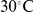 and at a constant pressure of 1 atm, both controlled with the weak-coupling scheme [41]. Translational and rotational motions were integrated with a 15 fs time step using the algorithm by Dullweber et al. [42]. Simulations were run with the Brahms program [43]. For these trial runs, systems of 864 and 2048 particles were used. The Lennard-Jones and inertial parameters were adjusted by seeking an incrementally closer agreement between the calculated bulk density and diffusion coefficients, and the corresponding experimental measurements. These adjustments were guided by simple rules based on physical intuition. Specifically, the system density could be increased, or decreased, by respectively increasing, or decreasing, the Lennard-Jones 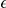 parameter (which controls interparticle attraction). The density could also be increased, or decreased, by respectively decreasing, or increasing, the Lennard-Jones 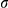 parameter (which influences the equilibrium distance between the sites). The diffusion properties of the model could instead be modulated by changing the particles' inertial features. By making the sites heavier, or lighter, the translational diffusion coefficient 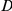 could be respectively decreased, or increased. Similarly, the rotational diffusion coefficient 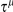 could be decreased, or increased, by respectively enlarging, or reducing, the particles' moment of inertia; since no experimental data are available for 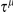, we targeted the corresponding results obtained by atomistic models [44]. The final set of optimized parameters is reported in table 1.

**Table 1:** Parameters of the ELBA water model.

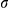	0.3 nm
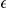	1.95 kJ/mol
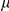	2.3 D
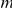	40 amu
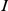	1 amu  nm 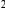
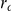	0.9 nm

The Lennard-Jones parameters 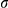 and 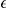 refer to equation 2, and the dipole moment 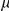 refers to equation 3. The parameters 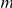 and 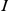 represent respectively the mass and the principal moment of inertia. The cutoff radius 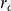 applies to both the Lennard-Jones (equation 2) and the dipole-dipole (equation 3) potentials.

To rigorously validate the ELBA water model, simulations were carried out with systems comprising 4000 sites, simulated at 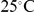 and 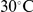 to allow a more thorough comparison with literature data. After equilibrating the systems for 1.65 ns, production runs were conducted for 3 ns. The results obtained are collected in table 2; for comparison, the table also reports experimental measurements and results obtained using various atomistic models. It can be seen that, as expected, the model results at 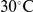 correctly reproduce the corresponding experimental measurements (chosen as parametrization targets). At 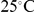, slight discrepancies can be noticed; these are not surprising, considering the simplicity of our model. In particular, corresponding to the temperature decrease from 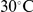 to 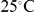, the ELBA model overestimates the experimental increase in density and it underestimates the decrease in the translational diffusion coefficient. While these discrepancies are not ideal, we believe our results remain satisfactory, especially considering that, overall, they are arguably as good as those obtained using typical atomistic models (table 2).

**Table 2:** Water physical properties.

	T	ELBA	Experiment [ref]	Atomistic models [ref]
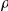 [g/cm 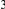 ]	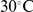	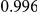	0.996 [151]	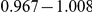 [44]
	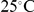	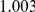	0.997 [151]	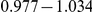 [40]
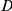 [ 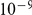 m 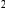 /s]	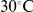	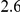	2.6 [152]	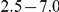 [44]
	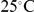	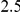	2.3 [152]	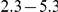 [40]
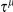 [ps]	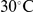	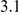	-	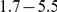 [44]
	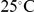	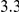	-	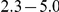 [40]

Symbols: 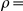
density, 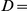
translational diffusion coefficient, 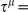
rotational diffusion coefficient. The ELBA results are characterized by relative errors of 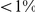.

To further assess the model, it would be useful to study the dielectric constant 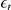. However, a reliable estimate of such a property can only be obtained when the long-range electrostatics of the model are explicitly accounted for [27], [45]. Since the ELBA force field (like most other CG models [1]–[4], [46], [47]) does not include long-range interactions, 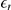 cannot be reliably quantified. As an approximation, it is however possible to alter the force field by including the long-range electrostatics, and calculate 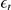 for such a modified model. We therefore sought to estimate 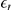 for bulk water by implementing a reaction field (RF) potential [23]. Unfortunately however, the introduction of the RF into the model caused simulation artifacts that prevented 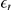 from being uniquely determined. In particular, we found the dielectric constant to be very sensitive to the choice of switching radius 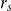 used to truncate the RF electrostatic interactions. For example, with 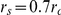 we obtained 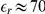, which is fairly consistent with the experimental value of 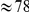 [48]. However, with a smaller switching radius 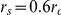 we obtained 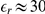, while with a larger radius 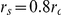 we obtained values of up to 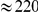. Full details of our RF calculations are reported in the Supporting Information S1. The observed sensitivity to the cutoff treatment of the RF potential was not completely unexpected, as cutoff-dependent results have been previously reported in the literature [44], [49], [50]. More generally, any model modification is bound to subtly alter the properties of the simulated system; for water simulations, the effects of variations in modeling details seem to be especially considerable and unpredictable [51]–[54]. It is evident that the ELBA water model is particularly sensitive to the additional RF potential; while this behavior is not ideal, it should be stressed that the only related limitation involves the inability to estimate the dielectric constant of bulk water systems. For all other purposes, the ELBA force field is meant to be used without a RF. It is also important to note that, despite not being able to provide a unique numerical estimate for the dielectric constant, we can still carry out an indirect and qualitative assessment of the dielectric behavior of the ELBA water model by considering the self-assembly properties of lipid-water systems. In particular, it will be shown in this paper that dispersions of lipids in water spontaneously self-aggregate into realistic membrane structures (either bilayers or inverse phases). Such phenomena rely on the dielectric screening of the lipid electrostatic interactions provided by the water dipoles. The ability of the ELBA membrane-water systems to self-assemble thus arguably indicates that the dielectric behavior of the water model is at least qualitatively correct.

The water phase behavior was also investigated, by simulating water systems at increasingly lower temperatures. Freezing was almost immediate at 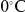, whereas at 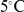 (and above) the system remained liquid (in particular, systems of 2048 sites were simulated for over 1 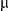
s); these findings are consistent with the well-known experimental behavior.

### Lipid models

In this work, we present and validate CG models for the following lipid species: dioleoylphosphatidylcholine (DOPC), distearoylphosphatidylcholine (DSPC) and dioleoylphosphatidylethanolamine (DOPE). These three lipids, while being structurally similar, are characterized by very different behaviors from one another; in fact, each of them can be considered representative of a different category amongst the most relevant for biological membranes. DOPC is one of the most typical lipids found in real biomembranes [55]–[59]. DOPC molecules in water spontaneously assemble into one of the most fundamental biological structure, that is, the fluid-phase bilayer membrane. DSPC is instead characterized, at biological temperatures, by spontaneous formation of solid (or “gel”) phases, which are also very important; for example, solid domains (sometimes called “rafts”) forming within fluid membranes are believed to play key roles in fundamental phenomena such as fusion, permeability, and protein regulation [60]. Structures formed by lipids in the gel phase are also prevalent in the skin [60]. Furthermore, solid-phase liposomes and nanoparticles can be engineered as drug delivery agents, DSPC being a common component [61], [62]. DOPE represents yet another fundamental category of lipids, characterized by the propensity to form “non-lamellar” (or “inverse”) phases. Interestingly however, various amounts of DOPE lipids are typically present in lamellar bilayer membranes; in fact, DOPE's (frustrated) tendency towards non-lamellar structures seems to provide an explanation for a large number of crucial processes, including the modulation of protein function [63]–[67].

**Figure 2:**
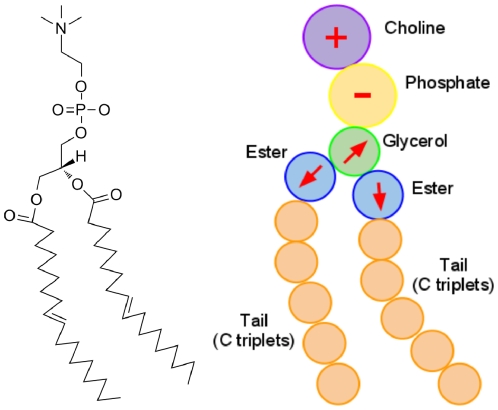
DOPC coarse-graining. The sketch on the left represented the chemical structure of a DOPC lipid. The corresponding CG model is depicted on the right. CG electrostatics are highlighted; they comprise positive (*“+” sign*) and negative (*“−” sign*) point charges in the headgroups, and point dipoles (*arrows*) in the glycerol and ester sites.

A sketch of the ELBA coarse-grain model for DOPC is reported in Figure 2, together with a standard atomistic representation. It can be seen that the DOPC molecule, which in reality comprises 138 atoms, is partitioned into 15 spherical CG sites. In particular, the lipid headgroup is represented by two sites, describing respectively the choline and phosphate moieties; the electrostatics are modeled through a positive point charge embedded in the choline site and a negative one in the phosphate site. The glycerol region is represented by a single particle embedded with a point dipole; the two ester groups, at the top of each tail, are also described by spherical sites embedded with point dipoles. The hydrocarbon tails are modeled with uncharged sites, each representing a consecutive triplet of carbon atoms (and associated hydrogens). The DSPC and DOPE models are derived straightforwardly from DOPC; in fact, these three lipids share an identical “backbone”. Therefore, the 15-site CG structure depicted in Figure 2 is used for all three lipid species considered in this study; each of the three models will then be specifically defined by different values assigned to the relevant parameters, as described in the “Parametrization” section.

### Interaction potentials

Intralipid (covalent) interactions between any bonded pair 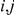 are modeled using the standard Hooke (harmonic) potential:
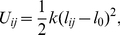
(4)where 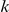 is a rigidity constant, 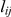 the actual bond length, and 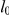 the reference bond length [24]. Consecutive triplets of sites 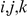 within the same lipid molecule also interact through the angular potential:
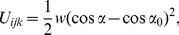
(5)where 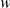 is a rigidity constant, 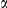 is the actual angle and 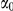 is the reference angle [33].

The repulsion-dispersion interaction between lipid sites is modeled through the shifted-force Lennard-Jones potential (also used in the water model) reported earlier in equation 2; here we specify the “mixing rules” used to assign the parameters 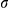 and 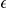 characterizing the interaction between a site of type 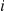 and a site of a different type 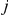. The parameter 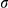 is defined by the standard Lorentz-Berthelot rule [23]:
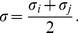
(6)Regarding the parameter 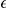, the following expression is used:
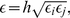
(7)where 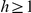 is a scaling constant. In particular, we set 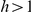 to account for the enhanced interaction energy of pairs capable of forming hydrogen bonds; for example, the Lennard-Jones interaction energy between a lipid phosphate site (containing several hydrogen-bond acceptors) and a water site (containing two hydrogen-bond donors) can be scaled up through 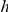; this factor therefore can be thought of as representing the (collective) “strength” of the corresponding hydrogen bond(s). For all the interactions that do not involve pairs capable of forming hydrogen bonds with each other, we set 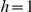, thus recovering the standard Lorentz-Berthelot mixing rule [23].

Electrostatic interactions are treated using classical expressions, slightly modified to address cutoff-related issues as already discussed above. Pair interactions between a point charge 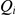 embedded in site 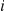 and a point charge 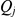 embedded in site 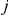 are modeled by a shifted-force Coulomb potential:
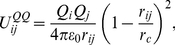
(8)where 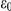 is the electric permittivity of vacuum, 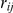 is the interparticle distance, and 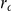 is the cutoff radius. Pair interactions between a point charge 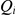 embedded in site 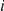 and a point dipole 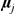 embedded in site 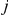 are modeled by a shifted-force charge-dipole potential:
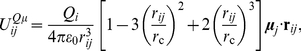
(9)where 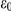 is the electric permittivity of vacuum, 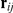 is the interparticle distance vector, 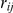 is the magnitude of 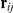, and 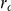 is the cutoff radius. Dipole-dipole interactions are modeled with the same potential adopted for the water dipoles (equation 3).

Lennard-Jones and electrostatic interactions are not computed for pairs of sites which are directly bonded to each other (this is similar to what is done in atomistic force fields, where intramolecular nonbonded interactions between atom pairs separated by one and two covalent bonds are neglected [25]).

The point dipoles embedded in the glycerol and ester sites are also subjected to a potential that restrains their orientation relative to the lipid molecule to which they belong, thus allowing the directionality of the corresponding atomistic charge distributions to be captured. In particular, the glycerol dipole is restrained to lie along the direction of the bond vector going from the glycerol to the phosphate site, whereas the ester dipoles are restrained to the bond vectors going from the ester to the adjacent tail site of the corresponding tail. Defining 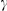 as the angle between a dipole vector and its “reference” bond vector (along which the dipole is restrained to lie), the following potential is therefore imposed:
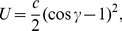
(10)where 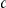 is a rigidity constant. In practice, this orientation-restraining interaction generates a torque on the dipoles to promote their alignment along the corresponding reference bond vectors.

The expressions of forces and torques corresponding to the potentials used in the ELBA force field are reported in the Supporting Information S1, together with additional details and explanatory sketches.

### Parametrization

The ELBA force field has been parametrized to reproduce the experimental measurements of some of the most fundamental properties of lipid membranes, namely, the area and volume per lipid [68], the dipole potential [13], and the spontaneous curvature [69]. The lipid area and volume represent possibly the most basic membrane structural features, and it is therefore essential to reproduce them as closely as possible. The membrane dipole potential characterizes the difference in the electrostatic potential between the hydrocarbon core and the outer water phase; it is receiving growing attention due to an increasing number of experimental studies showing its involvement in crucial biological processes [8]–[11], [13], [70]. The (monolayer) spontaneous curvature is also extremely important, as it controls phase stability and membrane fusion [71], and it affects the function of many membrane proteins [63], [67], [72]–[74].

Several force field parameters could be fixed at the outset. The masses of the CG sites were assigned considering the corresponding groups of atoms, as described elsewhere [5]. The moments of inertia of the glycerol and ester dipoles were set to 10 amu nm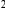, a value chosen intuitively as 10 times the moment of inertia of our water model. While we could have considered more elaborate parametrization procedures, it is important to remember that thermodynamic averages are not affected by inertial features [75], [76]. In our specific case, changes in the moments of inertia would only alter the rotational dynamics of the corresponding point dipoles. Therefore, the average values of the thermodynamic properties investigated in this study are not influenced by the specific values assigned to the moments of inertia of the lipid sites. The magnitude of the charges in each of the headgroup sites was set to 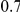 e, as in our previous model [5], [6]. The rigidity constant of the Hooke potential (equation 4) was set to 1260 kJ/(mol nm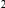) for all bonds; this value is within the range typical of CG lipid models [5], [6], [47]. Regarding the angular potential (equation 5), we chose for all angles a rigidity constant of 30 kJ/mol, a value which is again within the typical range of alternative CG force fields [47], [77]. The reference angles for the lipid tail sites could also be determined at the outset by analogy with previously reported CG models [6], [47], [78]. In particular, in the DOPC and DOPE models, the triplets of CG sites centered on the second tail site from the top (Figure 2) were assigned a reference angle of 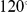; such a value mimics the tail kink imposed by the underlying cis-unsaturated double bonds [6], [47]. All other reference angles involving tail sites were set to 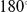 [47], [78].

The remaining parameters were determined through incremental refinements on the basis of the results obtained from trial simulations; this approach is similar to that adopted for the parametrization of the water model (as reported earlier in this paper) and of our previous lipid force field [5], [6]. We first focused on DOPC. Preliminary, unrefined parameters were set by intuition and by considering typical values used in published CG force fields [5], [6], [47], [77]. Trial simulations involved bilayer systems comprising 128 lipids and 4232 water molecules (corresponding to the experimental “full hydration” level of 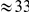 water sites per lipid molecule [68], [79], [80]). The temperature and pressure were maintained at 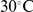 and 1 atm, respectively. These conditions reproduce those of experiments on fully-hydrated multilamellar systems in the liquid-crystalline phase [68], [79], [80].

To optimize the lipid volume (and hence the system density), we could tune the Lennard-Jones parameters in a similar way as previously described for the water parametrization. The lipid area was found to be particularly sensitive to the Lennard-Jones scaling factors 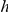 used to model hydrogen bonding (equation 7). For DOPC, we introduced three such scalings, to mimic the hydrogen bonds known to exist between the following site pairs: water-phosphate, water-glycerol and water-ester. As is intuitive, it was observed that larger values of the scaling factors 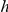 favored the partitioning of increasing amounts of water into the bilayer region comprising the phosphate, glycerol and ester particles, thus causing an expansion of the lipid area. The specific values of 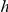 were tuned to approximate the relative strengths of the underlying hydrogen bonds as estimated by atomistic simulations. For PC lipids, it was found that the hydrogen bonds between water and the lipid phosphate group are stronger than those between water and the carbonyl oxygens of the ester regions, while the hydrogen bonds between water and the ether oxygens (which belong to the glycerol site in our CG representation) are weakest [81]–[83]. Further adjustments were performed by tuning the reference length 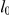 of the Hooke potential (equation 4). It was noticed that both lipid volume and area could be increased, or decreased, by respectively increasing, or decreasing, the value of 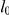. We eventually settled on the assignment of reference bond lengths through the general formula:
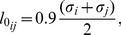
(11)where 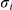 and 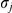 are the Lennard-Jones diameters of the two sites involved in the bond.

To reproduce the experimental spontaneous curvature [84], [85], we refined further the Lennard-Jones 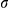 parameters to tune the relative size of the headgroup and tail sites, as in our original model [6]. In fact, the spontaneous curvature is a property that quantifies the monolayer tendency to bend. This tendency is related, at least in part, to the lipid shape (as is intuitive); for example, large headgroups and short tails normally increase the propensity to curl away from the water phase (forming for example micellar structures), whereas small headgroups and long tails tend to increase the desire to bend towards the water phase (forming “inverse” phases). Regarding the dipole potential, its experimental estimate [86], [87] could be reproduced by adjusting the magnitude of the glycerol and ester point dipoles, and the rigidity of the dipole orientation-restraining potential (equation 10).

The parameter set obtained for DOPC was subsequently extended to DSPC and DOPE. DSPC is structurally very similar to DOPC, the only difference being that while DOPC has a double-bond, or unsaturation, in the middle of each of the two tails, DSPC contains exclusively (saturated) single-bonds. The DSPC model could therefore be derived straightforwardly from the DOPC model by simply resetting the unsaturated reference angle of 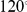 to the value of 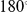 (already used for all the other tail sites corresponding to saturated bonds). Regarding DOPE, its parameter set could also be obtained by simply re-using most of the DOPC parameters. In fact, DOPC and DOPE are structurally identical apart from the terminal headgroup moiety, where the DOPC choline group is replaced in DOPE by a smaller amine moiety. In the ELBA representation, the CG site modeling the amine group in DOPE accounts for three “heavy” (non-hydrogen) atoms, that is, one nitrogen and two carbons. We therefore decided to model the amine using the same Lennard-Jones 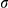 parameter as the “tail” type, as this type is also representing three heavy atoms (three carbons), and hence it is reasonable to assume a similar size. Regarding the Lennard-Jones interaction energy parameter 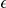, an additional important feature to consider for DOPE is that the amine group is known to feature three hydrogen bond donors, that can form hydrogen bonds with the phosphate, glycerol and ester groups, as well as with water. In the ELBA force field, this is captured by scaling up 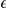 (see equation 7) for the relevant hydrogen bond pairs. These scaling factors 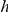 were tuned to reproduce the experimentally measured lipid area of DOPE lamellar systems [88]. The individual values of the 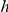 factors for DOPE were set to approximately reproduce the relative strengths of the underlying hydrogen bonds as determined by atomistic simulations, which indicated that the DOPE amine group forms the strongest hydrogen bonds with phosphate and ester-carbonyl groups [89], [90].

The final optimized parameter set, used for all the calculations presented in the remainder of this paper, is reported in table 3.

**Table 3:** Parameters of the ELBA force field.

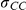 , 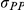	0.52 nm
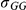 , 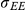	0.46 nm
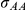 , 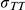	0.45 nm
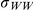	0.30 nm
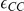 , 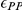	6.0 kJ/mol
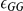 , 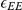	4.0 kJ/mol
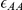 , 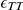	3.5 kJ/mol
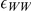	1 kJ/mol
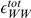	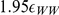
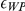	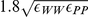
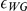	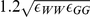
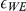	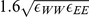
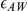	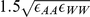
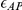	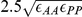
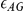	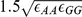
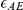	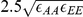
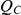 , 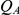	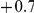 e
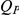	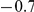 e
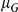	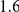 D
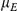	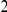 D
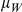	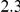 D
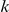	1260 kJ/(mol nm 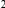 )
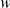	30 kJ/mol
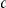	10 kJ/mol
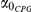 , 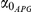	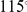
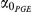	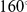
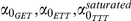	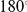
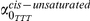	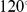
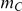 , 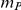	90 amu
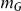 , 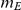	62 amu
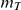 , 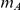	42 amu
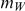	40 amu
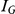 , 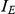	10 amu nm 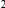
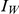	1 amu nm 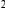

Subscripts 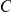, 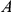, 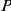, 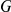, 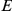, 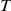 and 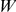 stand for the site types *choline*, *amine*, *phosphate*, *glycerol*, *ester*, *tail* and *water*, respectively. Lennard-Jones cross-terms are calculated by the standard Lorentz-Berthelot rules [23] except for increased 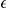 terms representing hydrogen bonding; in particular, 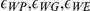, 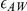, 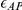, 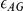, 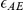 are set as reported in the table. Charges and dipoles are identified by 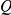 and 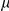; cross terms are obtained via standard electrostatic formulae [35]. The rigidity of the Hooke harmonic potential (equation 4) is identified by 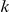; reference lengths are set to 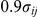. The rigidity of the angle potential (equation 5) is identified by 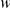; reference angles 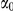 are reported for the relevant triplets of sites. The rigidity of the orientation-restraining potential (equation 10) is 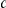. Masses and principal moments of inertia are identified by 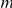 and 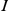, respectively.

### Simulation details

All simulations described in this paper were conducted using the molecular dynamics software Brahms [43]. The equations of motion were integrated using the algorithm by Dullweber et al. [42]. The integration timestep was 15 fs; this value was chosen empirically as a good compromise between the desire to use a large step size (to maximize sampling) and the need to satisfy energy conservation (the results of test runs using timestep values from 5 fs to 20 fs are reported in the Supporting Information S1). Pressure and temperature were controlled using the weak-coupling scheme [41]. The pressure was maintained at 1 atm in all simulations, with time constant 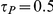 ps and isothermal compressibility 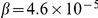 atm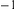. Lipid and water temperatures were coupled separately, with equal time constant 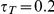 ps; for sites embedded with a point dipole, translational and rotational degrees of freedom were coupled independently. Systems were simulated at various temperatures, as specified in the next paragraphs. The cutoff radius for both Lennard-Jones and electrostatic water-water interactions was 0.9 nm, whereas all other nonbonded cutoff radii were set to 1.2 nm (as in previous work [5], [6]). The net mass center velocity of the entire system was removed at every step, as is commonly done to prevent possible drifting of the system and violation of energy equipartition [91]. To avoid artifacts in the evaluation of lipid diffusion [92], [93], the net lateral velocity of each of the two monolayers was also removed at every step.

Several simulations were conducted, as described in the following sections; each of these runs was carried out on a single processor. Thanks to the availability of a sufficient number of processors, all simulations could be run concurrently. Specifically, calculations were run on a supercomputer [94] equipped with Intel's 2.27 GHz Nehalem processors, as well as on desktop machines equipped with Intel's 3.33 GHz Xeon and 2.83 GHz Core2 processors.

### Preassembled systems

Simulations of preassembled bilayers were set up, for each of the three species considered (DOPC, DSPC, DOPE), at different sizes, hydration levels and temperature. Initial coordinates for hydrated bilayers were generated by adapting algorithms for standard cubic lattices [33]. The initial dimensions of the simulation regions were chosen to match the lipid area and volume, and the hydration level, of corresponding experimental systems [68], [79], [80], [88], [95], [96]. Initial velocities were assigned corresponding to the desired temperature [33]. The systems were then equilibrated in stages. The first stage involved running the systems at constant volume and desired temperature, for a few thousand steps, using incremental timesteps from 0.015 fs to 15 fs; this procedure allows gradual relaxation and initial convergence of energies, pressure and temperature. In the second stage, the systems were run for 1.5 ns at constant volume and temperature. The third and final equilibration stage involved simulating each system at constant pressure and temperature for 15 ns. The pressure was controlled by semi-isotropic volume scaling, meaning that the normal and tangential components of the pressure tensor were regulated separately. In particular, the pressure along the 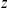-axis, that is, along the direction normal to the interface, was controlled by rescaling the 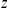-dimension of the simulation region, whereas the tangential pressure was controlled by rescaling the 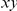 area, with the constraint that the interface remained a square. “Production” simulations were subsequently run for different lengths of time; a summary of these runs is reported in table 4. The final “wall clock” computation times for each simulation ranged from 3 to 6 weeks.

**Table 4:** Summary of simulations of preassembled bilayer systems.

Identifier	Lipid species	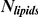	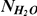	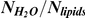	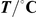	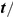 ns
A	DOPC	128	4232	33.06	30	1500
B	DOPC	512	16810	32.83	30	300
C	DOPC	1058	34848	32.93	30	150
D	DOPE	128	1152	9	22.5	1500
E	DOPE	512	4608	9	22.5	300
F	DOPE	1058	9522	9	22.5	150
G	DSPC	128	1536	12	30	1500
H	DSPC	128	4232	33.06	60	1500

Abbreviations: 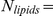
number of lipid molecules, 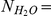
number of water molecules, 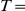
temperature, 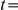
simulation time (excluding equilibration).

### Self-assembly runs

To study the self-assembly process, we set up a number of simulations starting from “random” mixtures of lipids and water (such dispersions were prepared by “disassembling” bilayer systems at high temperature, as described elsewhere [5]). Simulation settings were the same as described previously, with one exception: for these runs, the pressure was controlled by anisotropic volume scaling, meaning that each of the three components of the pressure tensor was regulated independently by rescaling the corresponding dimension of the simulation region.

## Results

In this section, we present and discuss the results obtained from the simulation of membrane systems modeled with the ELBA force field. Numerical results are reported in the form *average* 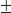 *standard error*; details on the error analysis performed can be found in the Supporting Information S1.

The calculation of a number of properties as a function of depth inside the membrane required subdividing the simulation regions into rectangular slabs perpendicular to the 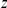 axis (normal to the bilayer). For each system, the number of slabs was chosen to yield a slab thickness 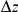 of 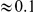 nm, the actual value of 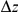 being recomputed at every step to account for the fluctuations of the 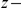
dimension of the simulation region. Such a procedure was adopted to calculate the profiles, as a function of 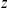, of the following properties: electron density, lateral pressure and electrostatic potential. To facilitate the interpretation of the profiles calculated, in the corresponding diagrams different regions across the system will be marked in italics, namely, the bulk *water* region, the lipid *headgroups* region and the lipid hydrocarbon *tails* core; moreover, approximate boundaries between these regions will be defined on the diagrams by vertical dotted lines. The profiles are presented as “raw data”, that is, no extra processing was done to smooth the curves; moreover, the data are not averaged over the two monolayers. Incidentally, we note that the approach used to calculate intramembrane profiles, which is standard in membrane simulations [76], [97]–[99], relies on the assumption that the bilayer is flat; we will show in the following that the validity of this assumption, especially for large systems, is questionable.

Simulation results will be primarily compared to experimental measurements; in some cases however, atomistic and CG models, and alternative theoretical approaches, will also be considered.

### Structural properties

From simulation of preassembled bilayers, the following structural parameters were calculated: area per lipid (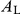), volume per lipid (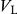), bilayer thickness (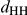), magnitude of headgroup dipole (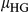), and orientation of headgroup dipole (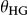). Moreover, from the fluctuations of 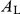, we estimated the area compressibility modulus (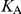). From the thickness 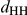 and the compressibility 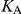 we then also computed the bilayer bending rigidity modulus 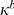. All these properties were calculated following standard procedures and formulae [6], [100]. The systems' structure was also analyzed in terms of the electron density distribution along the direction normal to the bilayer plane; approximate electron locations were defined by assigning to the mass center of each CG site all the electrons of the corresponding group of atoms, taking care that the total number of electrons per lipid in the models equals the real value (434 for DOPC, 438 for DSPC and 410 for DOPE).

**Figure 3:**
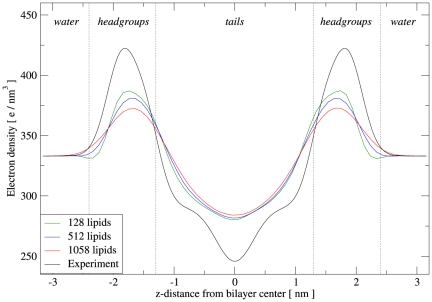
DOPC electron density profiles. The distributions calculated from simulations of the ELBA model are superimposed on the experimental profile obtained by Liu and Nagle [101]. The simulation curves refer to runs A, B and C.

**Figure 4:**
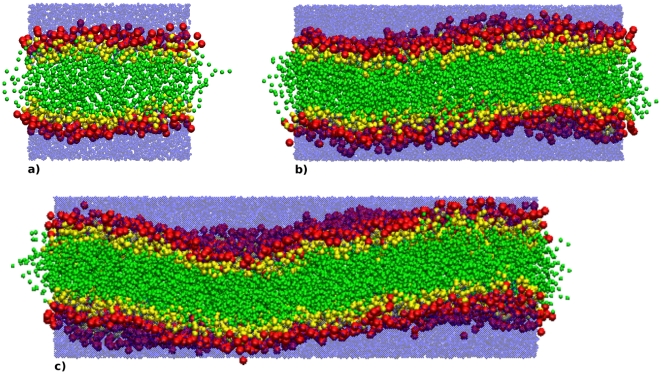
Snapshots of DOPC bilayers. Simulation snapshots: a) run A (128 lipids+4232 waters), b) run B (512 lipids+16810 waters), c) run C (1058 lipids+34848 waters). Choline and phosphate sites are red, glycerol and ester sites are yellow, tail sites are green and water sites are transparent blue.

The structural parameters calculated from our simulations of fully-hydrated DOPC bilayers at 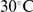 are collected in table 5. It can be seen that the experimental area and volume per lipid, which were chosen as parametrization targets, are correctly reproduced. The remaining parameters obtained from simulation show some discrepancies with respect to the experimental data; however, given that these properties were not parametrized for, the agreement is overall satisfactory. The electron density profiles are displayed in Figure 3, together with a corresponding experimental curve [101]; the calculated profiles are qualitatively realistic, although the magnitudes of the headgroup maxima and the central minimum are somewhat underestimated. It is also interesting to observe a slight size dependence; in particular, systems of increasing size show slightly wider headgroup peaks of lower magnitude. We believe that this is an artifact of the profile calculation method, rather than a “true” size dependence of the model (which would obviously be problematic). In fact, the calculation of the electron density profile relies on the assumption that the bilayer is perfectly flat. This condition is arguably met by small systems (e.g., less than 100 lipids), as undulations are suppressed; therefore, at any specific depth across the system, there is a well-defined, homogeneous layer, which is captured accurately by the corresponding rectangular slab used in the profile calculation. However, larger membrane systems do exhibit undulations, whose amplitude grows with the size of the simulated system; this can be qualitatively seen in Figure 4. The presence of undulations in larger bilayer is intuitively expected to “perturb” the content of each slab used in calculating the profiles, so that the resulting slab averages are “blurred” by the contributions of adjacent layers. Clearly, this effect is expected to increase with the size of the system (as seen in Figure 3). A related issue involves the estimation of the lipid area 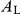. From simulation, 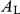 is typically obtained as 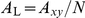, with 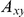 the total area of the 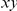 plane and 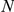 the number of lipids in each monolayer; such an expression yields the “projected” area per lipid, which is expected to decrease with increasing system size, again because of undulations. From our results (table 5), it can be seen indeed that the projected lipid area slightly decreases from the smallest to the largest system. Further detailed evidence of the issues discussed here can be found in a recent publication [102].

**Table 5:** Structural parameters of fluid-phase DOPC bilayers at full hydration.

Parameter	Run A (128 lipids)	Run B (512 lipids)	Run C (1058 lipids)	Experiment [reference]
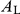 [Å 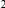 ]	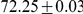	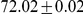	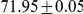	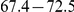 [68], [79], [80], [101], [153]
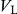 [nm 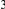 ]	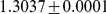	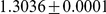	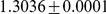	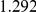 [85], 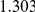 [80]
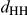 [nm]	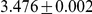	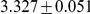	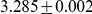	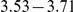 [79], [80], [101]
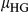 [D]	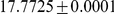	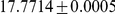	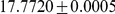	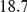 [154] b
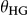 [deg]	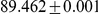	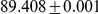	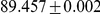	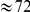 [106] b
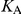 [dyn/cm]	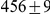	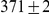	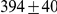	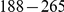 [79], [80], [100]
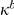 [k 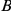 T]	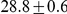	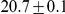	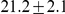	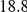 [80], 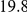 [101], 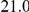 [100]

b Fluid-phase DPPC.

Regarding DOPE, we simulated preassembled bilayers at a temperature of 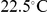 and with a hydration level of 9 waters/lipid; under these conditions, DOPE is experimentally observed to form bilayers [88]. Table 6 collects the results of our calculations together with the available experimental measurements. It can be noticed that area and volume per lipid are satisfactorily reproduced. As for the remaining structural parameters, unfortunately to our knowledge there are no corresponding experimental (or simulation) data reported in the literature. The electron density profiles can be found in the Supporting Information S1. Unsurprisingly, the size dependence effects noted above for the DOPC systems can be observed also for the DOPE results.

**Table 6:** Structural parameters of DOPE bilayers, 9 waters/lipid, 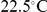.

Parameter	Run D (128 lipids)	Run E (512 lipids)	Run F (1058 lipids)	Experiment [reference]
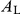 [Å 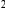 ]	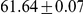	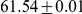	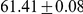	60 [88]
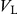 [nm 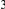 ]	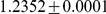	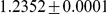	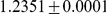	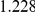 [133], 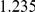 [85]
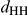 [nm]	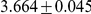	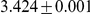	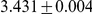	*not available*
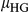 [D]	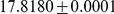	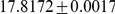	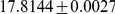	*not available*
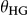 [deg]	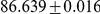	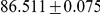	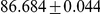	*not available*
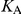 [dyn/cm]	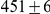	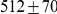	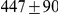	*not available*
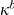 [k 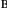 T]	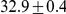	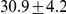	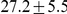	*not available*

**Figure 5:**
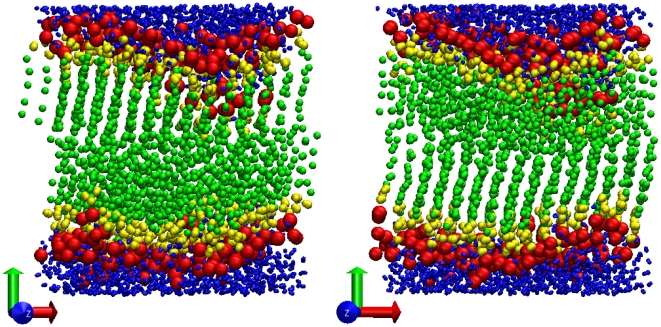
Gel-phase DSPC bilayer. Final snapshots from a simulation of a DSPC lipids bilayer at 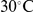 (run G).

**Figure 6:**
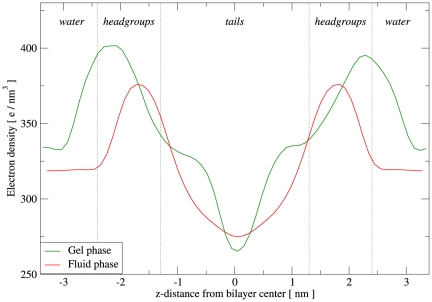
DSPC electron density profiles. The “gel phase” curve refers to the simulation at 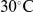 (run G), while the “fluid phase” curve refers to the simulation at 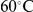 (run H).

The DSPC model was tested by running two simulations under different conditions, to reproduce the different phases characteristic of this lipid species. In fact, DSPC membranes are observed experimentally to form solid (gel) bilayers at temperatures below 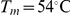 [103], [104]; above 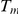, DSPC forms fluid-phase bilayers. One simulation was therefore run under “gel phase conditions”; in particular, run G was conducted at 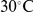 and 12 waters/lipid (as in corresponding experiments [105]). Example simulation snapshots from this system are presented in Figure 5; the two snapshots, taken at different angles, highlight the presence of tail ordering, a typical feature of gel phases. To quantify tail ordering, we calculated the segmental order parameter [106]:
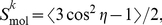
(12)where 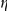 is the instantaneous angle between the 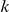-th bond along the tail and the direction normal to the bilayer plane (the 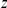-axis in our case). The angular brackets indicate averaging over the simulation time. By further averaging over the four bonds in each lipid tail, and then over all lipid molecules, we obtained a global tail order parameter 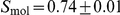. By definition, 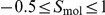; in particular, 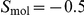 indicates alignment parallel to the bilayer plane, 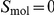 indicates random orientation and 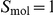 indicates alignment parallel to the normal to the bilayer plane. The calculated value of 0.74 is therefore rather large, and reflects a high degree of order; this is consistent with the “solid ordered” nature of gel-phase bilayer systems. A simulation under “fluid phase conditions” was also carried out (run H); the temperature was set to 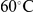, and the hydration level to 33 waters/lipid, consistent with corresponding experiments [96]. Visual inspection of run H revealed qualitative features common to fluid-phase bilayers (an example snapshot is reported in the Supporting Information S1). The global tail order parameter 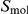 for this system was 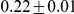; this value, much lower than that obtained for the gel-phase system, indicates substantial disorder, as expected for a fluid-phase membrane. The calculated electron density profiles of both gel and fluid phase DSPC are displayed in Figure 6. It can be seen that the separation distance between the headgroup peaks is larger for the gel than for the fluid system, indicating that the gel bilayer is thicker than the fluid one; this finding, related to tail ordering in the gel phase, is consistent with experimental observations [60], [68]. All the structural parameters obtained from our simulations are presented in table 7, together with the available experimental measurements. It can be seen that the calculated area per lipid under gel phase conditions is very close to the experimental value. For the fluid phase run, the calculated area is instead somewhat higher than the experimental value, while the thickness is slightly smaller. Regarding the area compressibility modulus 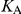 and the bending rigidity modulus 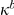, to our knowledge there are no data in the literature to use for comparison. However, we can use reported measurements on dimyristoylphosphatidylcholine (DMPC) and dipalmitoylphosphatidylcholine (DPPC) bilayers. These lipids are very similar to DSPC; they share the same headgroup and glycerol-ester regions, and are made of fully-saturated tails. The only differences involve the slightly shorter tails; in fact, while DSPC has 18 carbons per tail, DPPC and DMPC have respectively 16 and 14 carbons. From table 7 it can be seen that, in terms of absolute magnitudes, the values calculated from our simulations are larger than the experimental figures for 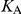, while they are similar for 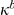. It is perhaps more interesting to look at relative values, because it is known experimentally that these elastic constants, when measured for gel-phase systems, are several times larger than the corresponding values for the fluid phase [107], [108]. In this respect, table 8 shows that our simulation data reproduce remarkably well the corresponding experimental results. Such large gel/fluid ratios for 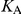 and 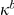 reflect fundamental (and intuitive) properties: upon transition from fluid-like to solid-like state, bilayers become substantially less compressible and more rigid.

**Table 7:** Structural parameters of DSPC bilayers.

	Gel phase @ 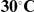		Fluid phase @ 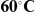	
	Run G	Experiment [ref]	Run H	Experiment [ref]
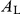 [Å 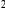 ]	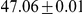	47.3 [105]	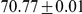	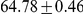 [96]
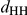 [nm]	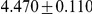	-	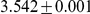	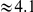 [96]
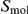	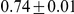	-	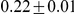	-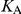 [dyn/cm]	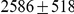	855^a^ [107]	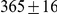	144^a^ [107]
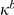 [k 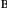 T]	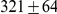	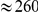 b [108]	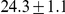	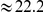 b [108], 37.1^b^ [155]

a DMPC.

b DPPC.

**Table 8:** Gel-to-fluid ratios for elastic properties of DSPC bilayers.

	ELBA model	Experiment [reference]
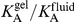	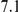	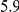 a [107]
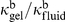	13.2	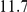 b [108]

a DMPC.

b DPPC.

To further confirm the phase states of the simulated systems, we also calculated the lipid lateral diffusion coefficients; results will be reported in the dedicated “Lipid diffusion” section below.

### Intramembrane pressure

The pressure distribution inside a membrane can be characterized through the “lateral pressure profile” [60], [69], [109], [110]. The lateral pressure profile is conventionally defined as the difference (as a function of depth inside the membrane) between the pressure tangential to the membrane plane and the pressure normal to the membrane plane. In symbols, defining the coordinate 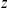 as perpendicular to the membrane plane, the lateral pressure profile can be written as 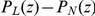, with 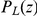 the average of the “lateral” components of the pressure tensor, that is, 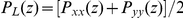, and 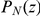 simply being the “normal” pressure, that is, 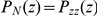. Positive values in the lateral pressure profile thus reflect underlying “outward” forces, wishing to expand the corresponding membrane plane. Conversely, negative values relate to “inward” forces with a tendency to compress the membrane area.

**Figure 7:**
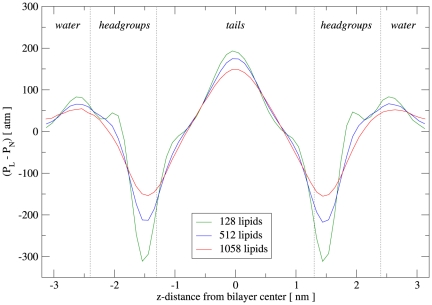
DOPC lateral pressure profiles. The curves refer to runs A (128 lipids), B (512 lipids) and C (1058 lipids).

We calculated the lateral pressure profiles from simulation following a standard methodology [6], [111]. The profiles obtained for DOPC are displayed in Figure 7. We note that the qualitative shape of the pressure distribution is consistent for all three curves; however, as noted earlier for the electron density profiles (Figure 3), there is a dependence of the magnitudes on the size of the simulation system. We believe that the cause of this effect lies in the calculation procedure, rather than in the model, as discussed previously. In particular, since increasingly large bilayers display undulations of increasing amplitudes, the “flat bilayer” assumption made in the profile calculation procedure is progressively undermined, resulting in a gradual “smoothing” of the profiles for larger and larger systems. This can be seen clearly in Figure 7, where the magnitudes of peaks and troughs for the (largest) 1058-lipid bilayer are lower than for the (smallest) 128-lipid system, with the 512-lipid featuring intermediate magnitudes. In general, it can be seen that the profiles are characterized by local maxima close to the headgroup-water interface; the molecular origin of these “membrane-expanding” forces reflect repulsive interactions of steric, electrostatic, and hydration nature [109]. Inside the headgroups region, close to the interface with the hydrocarbon tails, the lateral pressure drops sharply, to negative values of large magnitude. These prominent troughs, indicative of “membrane-contracting” forces, originate in the interfacial tension, whereby the bilayer hydrocarbon core wishes to minimize its exposure to the outer hydrophilic environment. The pressure then rises again upon entering the hydrocarbon tail region, forming a broad peak centered in the middle of the bilayer. The repulsive forces underlying the central pressure peak are believed to originate from entropy losses; the tight molecular packing in the membrane core induces the lipid tails to stretch (thus losing entropy relative to isolated “free” tails), ultimately leading to significant inter-tail repulsion [112], [113].

Unfortunately, no experimental data are available for a quantitative assessment of the profiles obtained with our model. In fact, the single experimental investigation of the DOPC pressure profile reported to date only provides qualitative data for part of the tail region [114]. A DOPC pressure profile was published for an atomistic model [97]; that profile is somewhat different from ours, especially in the tails region. While atomistic models are generally expected to be more accurate than CG models, in this case there is some evidence to the contrary. In fact, the results for the spontaneous curvature, an experimentally-measurable parameter obtained from integrating the pressure profile (see next section), are more realistic for our model. In particular, the spontaneous curvature of 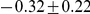 nm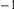 calculated for the atomistic model [110] is somewhat inconsistent with the experimental range of 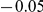 to 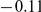 nm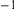 [84], [85]; our results are instead inside the experimental range (see next section). It should be noted that the lateral pressure profile is a slowly converging property, and hence it is more difficult to obtain reliable results with an atomistic model, due to the limits on the accessible simulation timescale.

**Figure 8:**
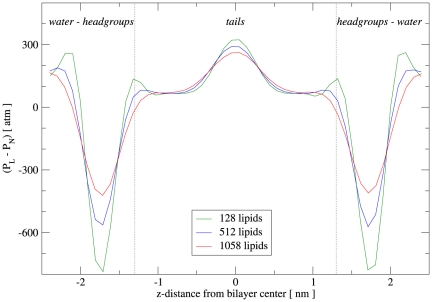
DOPE lateral pressure profiles. The curves refer to runs D (128 lipids), E (512 lipids) and F (1058 lipids).

Regarding DOPE, the calculated pressure profiles are displayed in Figure 8; it can be noticed that these curves share the main qualitative features highlighted previously for DOPC. It is also clear that the size of the system affects the calculated magnitudes in the same way as discussed above for DOPC. A quantitative assessment of the curves obtained is difficult to carry out, because to our knowledge there are no published DOPE pressure profiles in the literature, neither from experiment nor from simulation. An indirect assessment is however possible through the analysis of the curvature constants derived from the pressure profile (see next section).

**Figure 9:**
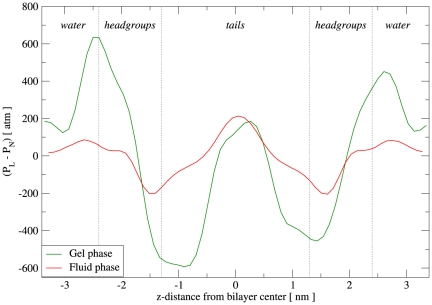
DSPC lateral pressure profiles. The “gel phase” curve refers to the simulation at 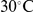 (run G), while the “fluid phase” curve refers to the simulation at 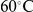 (run H).

The pressure profiles for DSPC, both in the gel and in the fluid phase, are reported in Figure 9. It is evident that, while the profiles are qualitatively similar (in terms of the number and location of the main peaks and troughs), the magnitudes involved are markedly different. In particular, the outermost peaks at the headgroup-water interface are characterized by pressures which are 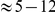 times larger for the gel than for the fluid phase. Regarding the main troughs, located around the headgroup-tail interface, the magnitudes of the gel phase curve are 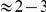 times larger than those of the fluid phase. Both profiles display similar magnitudes for the central peak. It can be noticed that the fluid phase profile is rather similar to the curves obtained for fluid phase DOPC (Figure 7), as intuitively expected. To our knowledge, there is no available pressure profile for DSPC in the literature (either experimental or from simulation), so it is difficult to quantitatively validate our results. An indirect assessment is again possible through the calculation of the spontaneous curvature; as discussed in the next section, the results obtained are qualitatively realistic. In terms of the pressure profile differences highlighted in the comparison between gel and fluid phases, our results are in qualitative agreement with the calculations for DPPC gel and liquid domains reported by Ollila et al. [115].

### Spontaneous curvature and elasticity properties

According to Helfrich's well-established theory [116], the surface curvature elastic energy per unit area can be defined as:
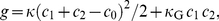
(13)with 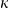 the bending rigidity, 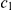 and 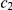 the (local) principal curvatures, 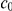 the spontaneous (or intrinsic) curvature, and 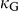 the Gaussian curvature modulus. The constants appearing in equation 13 can be derived from the first and second integral moments of the pressure profile [112]. Equation 13 is valid for the entire bilayer as well as for monolayers; when considering flat membranes (as in our case), the curvature elastic parameters are typically evaluated for monolayers. In the following, “monolayer properties” will be indicated by the use of the superscript 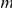.

Defining the lateral pressure profile as 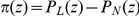, the first integral moment 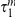 is:
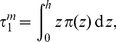
(14)and the second integral moment 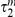 is:
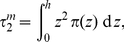
(15)where 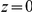 at the center of the bilayer and 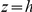 in the water phase [112]. In practice, the integrations were carried out over each of the two monolayers, with 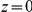 and 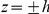, 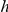 being half the 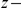
dimension of the simulation region; the results reported here represent averages over the two monolayers of each bilayer system.

From the first moment (equation 14) it is possible to calculate the spontaneous curvature as 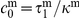, 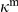 being the monolayer bending rigidity modulus. The monolayer bending rigidity modulus can be simply obtained from the bilayer modulus as 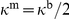 [71]. Combining the first and second moment (equations 14 and 15) it is possible to evaluate the Gaussian curvature modulus as 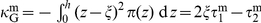, 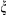 being the distance to the *pivotal surface*, defined as the surface at which there is no change in the molecular cross-sectional area upon bending [71]. The pivotal surface has been experimentally located close to the polar/apolar interface [117]. Considering the lateral pressure profiles (Figures 7, 8 and 9), we assume the polar/apolar interfaces of the two monolayers to be located at the two global minima of the curve, corresponding to the two main lateral pressure troughs. In fact, it is reasonable to claim that the pressure profile troughs identify the regions of largest surface tension, which develops at the hydrophobic/hydrophilic interfacial regions. Hence, by computing the half-distance between the global minima of the pressure profile, we obtained 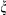 for each simulated system.

The curvature elastic parameters obtained for DOPC are collected in table 9, together with corresponding experimental data; it can be seen that the results from our simulations fall inside the experimental ranges, for all bilayer sizes considered. For DOPE, results are collected in table 10; the values obtained are rather close to the available experimental measurements, although the spontaneous curvature is somewhat less negative than the experimental data, while the first integral moment is marginally more negative. Regarding DSPC, we have calculated the curvature properties from both simulations in the gel and fluid phase; results are reported in table 11. To our knowledge, there are no comparable experimental or simulation data available in the literature. We note however that the absolute values of the monolayer spontaneous curvature are very low, both in the gel and in the fluid phase; these results are qualitatively realistic, as they are consistent with the experimental observation that DSPC forms bilayer structures (as opposed to micelles or inverse phases) [96], [103], [105].

**Table 9:** Curvature elastic parameters of fluidmphase DOPC bilayers at full hydration.

	Run A (128 lipids)	Run B (512 lipids)	Run C (1058 lipids)	Experiment [reference]
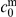 [nm 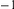 ]	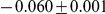	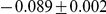	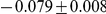	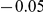 to 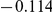 [84], [85] a
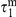 [k 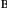 T/nm]	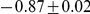	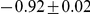	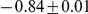	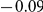 to 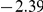 [80], [84], [85], [100]
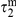 [k 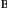 T]	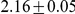	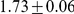	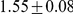	*not available*
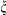 [nm]	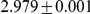	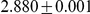	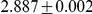	*not available*
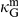 [k 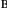 T]	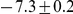	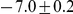	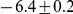	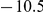 to 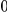 [100], [117]

a Szule et al. [84] report an estimate of 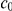 from 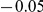 to 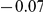 nm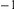 at 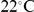.

**Table 10:** Curvature elastic parameters of DOPE bilayers, 9 waters/lipid, 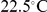.

	Run D (128 lipids)	Run E (512 lipids)	Run F (1058 lipids)	Experiment [reference]
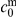 [nm 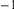 ]	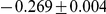	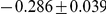	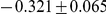	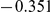 [85], 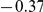 [156]
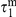 [k 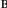 T/nm]	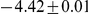	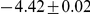	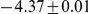	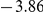 [85]
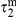 [k 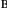 T]	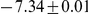	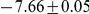	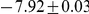	*not available*
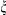 [nm]	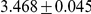	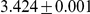	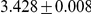	*not available*
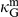 [k 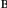 T]	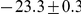	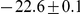	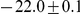	*not available*

**Table 11:** Curvature elastic parameters of DSPC bilayers.

	Run G (gel phase @ 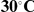 )	Run H (fluid phase @ 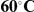 )
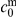 [nm 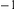 ]	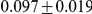	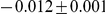
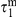 [k 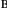 T/nm]	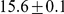	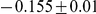
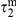 [k 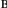 T]	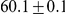	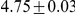
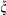 [nm]	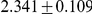	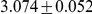
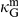 [k 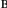 T]	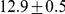	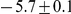

### Electrostatic potential

Considering a coordinate 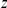 running along the 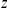-axis (which is, by convention, perpendicular to the membrane interfacial plane), the electrostatic potential profile 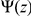 was obtained according to:
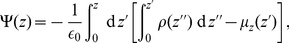
(16)with 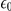 the permittivity of free space, 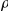 the charge density of the system, and 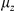 the projection of the sum of the point dipole vectors along the 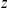-axis [118]. In our systems, 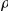 is the total charge density of the lipid headgroup charges, and 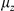 is the 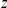-projection of the vector obtained by summing up the water, glycerol and ester point dipoles.

The intramembrane electrostatic potential is a very difficult property to study experimentally [13]; to our knowledge, depth-dependent measurements of 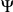 have never been reported. However, various experimental techniques have been employed to obtain (indirect) estimates of the “dipole potential” 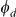, defined as the difference between the potential in the hydrocarbon core with respect to that in the water phase [119], [120]. While accurate and direct measurements are deemed practically impossible [13], the various estimates reported for fluid-phase PC bilayers are consistent in characterizing 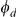 as positive, with a magnitude of 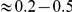 V [86], [87], [120]–[122]. The values of 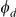 calculated from our simulations are reported in table 12; it can be seen that the results obtained for fluid-phase DOPC and DSPC (runs A, B, C, H) fall inside the reported experimental range. For DOPE (runs D, E, F) and gel-phase DSPC (run G), we are not aware of any comparable value reported in the literature.

**Table 12:** Dipole potential.

Run	A	B	C	D	E	F	G	H
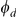 [V]	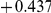	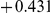	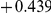	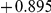	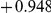	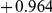	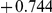	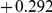

The run details are reported in table 4. Values are characterized by a relative error of 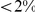.

**Figure 10:**
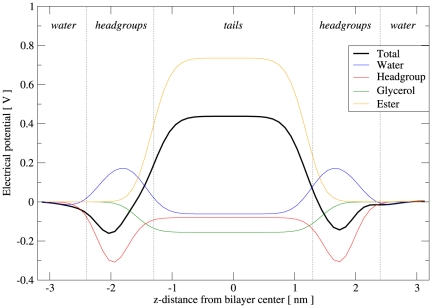
DOPC electrostatic potential profile. The total transmembrane potential is plotted together with the individual contributions of the various charged species in the system. The data were obtained from run A.

Regarding the full electrostatic potential profile 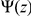, our results for DOPC are reported in Figure 10; the total profile is shown together with single-site contributions. It can be seen that the main contributors to the potential are the ester dipoles, whereas the effects of the other charged species are comparatively minor. While no comparable experimental data are available, the different contributions to the electrostatic potential for fluid-phase PC bilayers have been calculated using atomistic models [123]–[126]. In those investigations, the main contributor to the dipole potential was found to be water; this is different from the result obtained with our model. The cause of this disagreement lies in the different orientational behavior of the water molecules in our model and in the atomistic simulations. In particular, the large water electrostatic potential observed in the atomistic systems derives from a strong preferential alignment of the water dipoles interacting with the lipid headgroups. In our simulation, we instead observe weaker orientational effects, which result in a much less pronounced contribution to the overall potential. Regarding the specific contribution of the glycerol-ester region, our data are qualitatively consistent with the results obtained in the all-atom simulations by Shinoda et al. [123], [124]. However, the glycerol-ester contribution to the overall potential is almost negligible in united-atom models [126].

Regarding 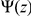 for DOPE and DSPC, the results from our simulations are reported in the Supporting Information S1; to our knowledge, no corresponding data exist in the literature.

### Lipid diffusion

We calculated the lateral diffusion coefficient 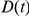 through the standard expression:
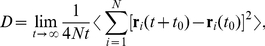
(17)where 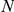 is the number of lipids, 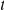 is the measurement time, 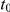 is the time at which a measurement starts, and 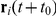 and 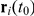 are the center of mass positions of lipid 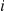 in the plane of the bilayer at times 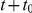 and 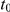. The angular brackets in equation 17 indicate an averaging over different starting times 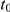. We calculated the diffusion coefficients for runs A, D, G, and H, considering a measurement time 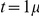
s. In particular, we selected 26 starting times 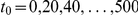 ns and carried out 26 corresponding diffusion measurements for 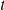 extending to 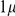
s. For the DOPC system (run A), characterized by a hydration level of 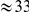 waters/lipid, we obtained a value of 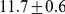 nm
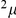
s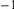; this result compares remarkably well with the experimental measurement of 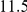 nm
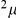
s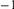 for a hydration level of 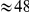 waters/lipid [127], and with the value of 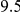 nm
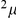
s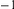 for a hydration level of 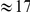 waters/lipid [128]. Regarding the DSPC systems, at 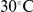 (run G), we obtained a very low value of 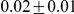 nm
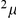
s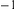, consistent with the bilayer being in the gel state. At 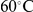 (run H), we obtained a value of 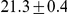 nm
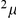
s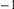, which can be compared favorably with the measurement of 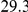 nm
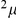
s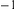 for fluid-phase DOPC at the same temperature [127]. As for the DOPE simulation (run D), we obtained a diffusion coefficient of 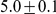 nm
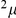
s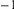; to our knowledge, the diffusion coefficient for DOPE bilayers has not been reported previously in the literature. The complete curves of the diffusion coefficient 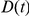 for the systems considered, as a function of the measurement time, can be found in the Supporting Information S1.

### Water permeability

By monitoring water particles that spontaneously cross the bilayer during the course of our simulations, it is possible to estimate the corresponding permeability coefficients 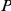 using Fick's first law of diffusion:
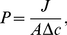
(18)where 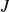 is the unidirectional flux of water, 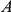 is the interface area, and 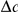 is the water concentration gradient. In this work 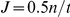, where 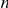 is the number of water particles that crossed the bilayer during the simulation time 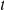, and the factor 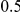 is used to obtained a single (average) unidirectional flux from the two opposing fluxes contributing to 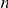. The interface area 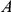 is the average 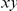 area of the simulation box (where the 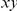 plane is parallel to the membrane plane by construction). The water concentration gradient is 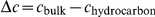, where 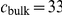 nm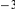 is the standard water concentration in the bulk phase and 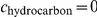 is the (negligible) concentration of water in the hydrocarbon core of the bilayer. The permeability coefficients calculated for our simulations are reported in table 13. For the DOPC systems, it can be seen that the agreement with the available experimental measurements is very good. Regarding DOPE, no experimental data were found in the literature. However, relevant experiments have been reported for other PE species, together with corresponding PC species characterized by the same tail length [129], [130]. A general finding from these experiments is that water permeates faster through PC than through PE membranes. This phenomenon is present in our simulations too; table 13 shows that the permeability coefficients for the DOPC bilayers are 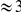 times larger than those for the DOPE systems. As discussed in the literature, the smaller interfacial area of PE bilayers compared to PC systems results in increased packing density in the tail region, which (as is intuitive) obstructs the flux of water, ultimately reducing the permeability coefficient [131]. The ratios between water permeability coefficients through PC and PE bilayers from our calculations and from the measurements reported in the literature are tabulated in the Supporting Information S1. Regarding DSPC, it can be seen from table 13 that the permeability coefficients from our simulations are larger than those obtained from experiments, both in the gel and in the fluid phase. However, our model correctly predicts the experimental finding that the water permeability coefficient through the lipid gel phase is 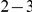 orders of magnitude lower than that through the lipid fluid phase. The ratios between water permeability coefficients through gel and fluid DSPC bilayers from our calculations and from experimental measurements are tabulated in the Supporting Information S1.

**Table 13:** Water permeability coefficient 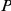 [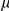
m/s].

Lipid	Simulation data (this work)	Experimental data [reference]
DOPC (fluid)	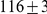 (run A), 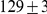 (run B), 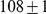 (run C)	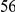 [157], 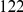 [131], 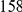 [130]
DOPE (fluid)	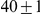 (run D), 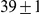 (run E), 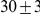 (run F)	*not available*
DSPC (gel)	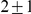 (run G)	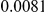 [129], 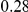 [129]
DSPC (fluid)	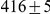 (run H)	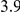 [129], 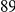 [129]

### Self-assembly

**Figure 11:**
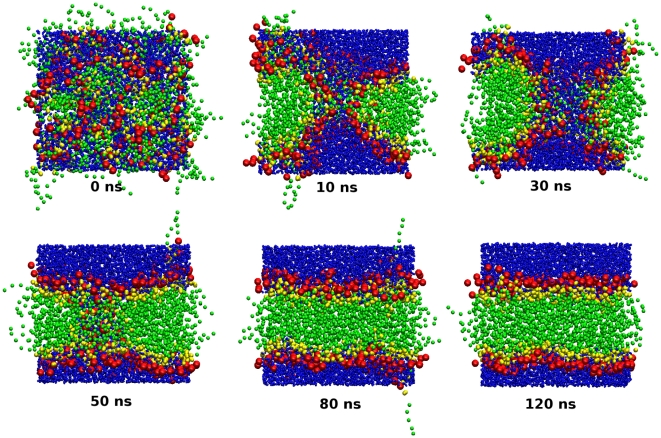
Self-assembly simulation snapshots. Trajectory snapshots from a self-assembly simulation of 128 DOPC lipid molecules and 4232 water sites.

Starting from “random” mixtures of DOPC lipids and water, we simulated the spontaneous self-assembly of stable defect-free bilayer structures; snapshots from a typical DOPC self-aggregation simulation are displayed in Figure 11. The initial stages of the process involve the separation of water and the formation of lipid clusters; this is followed by the formation of a water pore, which eventually disappears, leaving a defect-free bilayer structure. The time-scale and the overall aggregation mechanism are comparable with self-assembly atomistic simulations reported in the literature [99], [132]. Incidentally, it is interesting to analyze self-assembled bilayers and compare them to preassembled systems. In fact, the prediction of bilayer properties from preassembled structures relies on the assumption that the systems simulated are at thermodynamic equilibrium; this is difficult to establish, because, for example, systems might be “trapped” in long-lived metastable states. Another assumption underlying the characterization of lipid bilayers from simulation involves the convergence of all properties of interest over the simulation time; again, this is difficult to rigorously prove, because of the possible existence of very long correlation times. The comparison of properties calculated from self-assembled bilayers with those obtained from corresponding preassembled structures offers a way to check the reliability and consistency of the calculations. We therefore simulated a self-assembled DOPC bilayer for an additional 750 ns, over which time we performed the same analysis carried out on the corresponding preassembled bilayer (run A). Reassuringly, the results obtained from the self-assembled system are consistent with those from the preassembled simulations; detailed comparisons con be found in the Supporting Information S1.

**Figure 12:**
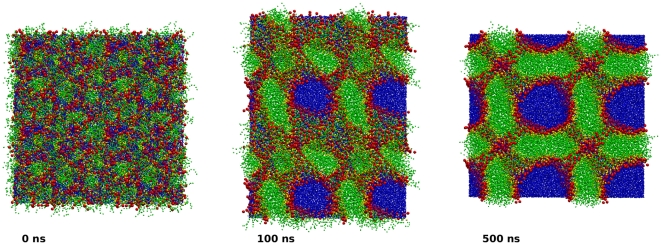
Self-assembly simulation snapshots. Trajectory snapshots from a DOPE-water system. To facilitate interpretation, each panel was prepared by juxtaposing four replicas of the main simulation region through their periodic boundaries. The main simulation region comprises 512 DOPE lipid molecules and 10890 water sites.

Regarding DOPE, the experimental phase diagram highlights a rather complex behavior [88], [133], [134]. In fact, for temperatures less than 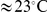 and for hydration levels between 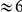 and 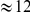 waters/lipid, DOPE forms a lamellar bilayer phase (which we simulated in runs D, E, F analyzed above). Outside these conditions, DOPE forms inverse phases. To study the formation of inverse phases, we conducted simulations of dispersions of DOPE and water at 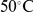 and 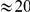 waters/lipid. Snapshots from a typical self-assembly run are reported in Figure 12. An initial quick phase separation leads to the formation of water columns, as can be noticed in the middle panel of Figure 12. The system subsequently stabilizes into a cubic-like structure (right panel of Figure 12); this configuration, while being an inverse phase, does not correspond to the hexagonal phase observed experimentally. While this is not ideal, it is important to stress that the DOPE parameters were developed to capture the main structural properties of lamellar phases, at low temperature and hydration, and hence it is not surprising that the model does not perform equally well under the substantially different conditions employed in the self-assembly runs. However, it could also be that the accessible simulation length (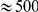 ns) did not allow the system to reach equilibrium. In fact, the final simulation snapshots show “defects”, in the form of clusters of lipid headgroups cross-linking the water columns; these structures might be indicative of metastable states.

## Discussion

The ELBA force field for coarse-grain molecular dynamics simulation of membranes has been presented. Specific models were validated for the DOPC, DOPE and DSPC lipid species. The force field also includes a new coarse-grain dipolar water model, which we developed by parametrizing the Stockmayer potential to represent individual water molecules.

A notable and unique characteristic of the ELBA CG model is that the Lennard-Jones interactions between different site types are simply treated through the standard Lorentz-Berthelot mixing rules [23] (with some adjustments to treat hydrogen-bonded pairs), as is common in atomistic models [24], [25]. This is different from alternative CG force fields, where every pair interaction involving different site types is specifically parametrized [46], [47], [77], [78]. The ability of our model to take advantage of the general and intuitive combination rules for the mixed Lennard-Jones interactions depends on the explicit treatment of the lipid electrostatics and the water dipoles, which interact realistically with each other through a relative dielectric constant of unity (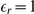, as in atomistic force fields). More precisely, we believe that such a description of the electrostatics is a necessary (though not sufficient) condition for the ability to use the Lorentz-Berthelot formulae in the ELBA model; in fact, it is most likely the explicit presence of the main electrostatics that allows the balance of forces between hydrophilic (polar) and hydrophobic (apolar) sites to be achieved without the need to deviate from the standard mixing rules for the Lennard-Jones interactions. This is advantageous, because the underlying physics is represented more faithfully. Moreover, transferability and extensibility of the model are expected to be facilitated; for example, new species can be added to the force field without the need to specifically parametrize mixed interactions.

Single-component bilayers were simulated under fluid phase conditions; the force field proved capable of reproducing experimental measurements for several fundamental properties, including structural parameters, curvature elastic constants, the electrostatic potential distribution, the lipid diffusion coefficient, and the bilayer self-aggregation process. A realistic gel phase was also obtained by simulating DSPC at a temperature below its experimental transition temperature.

A comparison of the lateral pressure profiles obtained for different lipids highlights large variations in the peak values (Figures 7, 8 and 9); in particular, the magnitudes characterizing the pressure profiles for DOPE and gel-phase DSPC are several times larger than those observed for DOPC. Our data thus show clearly and quantitatively a pronounced sensitivity of the internal pressure distribution to changes in the lipid composition of bilayers. This is a crucial phenomenon, with far-reaching consequences; by acting as an amplifier of changes in the lipid environment, the lateral pressure profile is thought to provide a fundamental mechanism for the regulation of membrane proteins [60], [67], [69], [135].

The simulation of bilayer systems of different sizes allowed us to highlight a size dependence in the calculation of the lipid area and generally of intramembrane profiles, which was explained in terms of membrane undulations not accounted for by the calculation procedures employed. The severity of the resulting artifacts was shown to correlate with the system size. Since the procedures that we employed are standard [76], [97]–[99], the problem highlighted in this paper is expected to generally affect membrane simulations, irrespectively of the specific modeling details. Furthermore, these artifacts are likely to become more severe in the future, because the growing computer power will allow larger membranes to be simulated (in general, increasing the size of simulated systems is desirable, to limit periodicity and finite-size effects). A more detailed assessment of this problem can be found in a dedicated study recently published by Braun et al. [102], where methods to address the problem are also proposed.

A notable and somewhat surprising aspect of the ELBA model is its ability to display a realistic dynamical behavior. This represents an improvement over alternative CG models, for which diffusion coefficients for lipids in the liquid phase have been reported to be four [47], [136] to one hundred [137] times higher than corresponding experimental measurements. In fact, most CG models are characterized by a smoother free energy landscape (with lower barriers) which induces faster kinetics. However, our CG approach represents an exception, as neither the results presented here nor those obtained with our previous models [5], [6] show faster lipid dynamics (at least for the lateral diffusion process). A possible explanation involves the correct diffusional behavior of our water model; due to its presence in the polar/apolar interface region of the bilayer, water might regulate the lipids' dynamics. Another contributing factor is probably related to the presence in the ELBA model of realistic electrostatic interactions (with 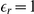), giving rise to a rougher free energy landscape compared to that of alternative CG models where such interactions are either absent [78], [136] or weakened by imposed artificial screening (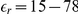 [47], [137]).

To quantify the computational efficiency of the ELBA model, we estimated the gain in sampling speed with respect to two standard atomistic approaches (united-atom and all-atom) by simulating membrane systems representing an equivalent number of lipid and water molecules. All details of these benchmarks are reported in the Supporting Information S1. Bearing in mind that performance comparisons involving different models and computer programs are inevitably uncertain and disputable, because of the many variables involved (system size, protocol parameters, hardware used, etc.), in the tests carried out the ELBA model proved respectively 15 and 200 times faster than equivalent united-atom and all-atom models.

In terms of limitations of our methodology, two aspects should be discussed. Regarding the force field, we believe that the only major issue involves the self-assembly of DOPE systems, which did not reproduce the experimental inverse hexagonal phase; rather, the formation of a pseudo inverse cubic phase was observed. Future efforts will be devoted to reconcile the model's results with the experimental findings. In particular, longer simulations will have to be conducted (to assess the attainment of equilibrium) and larger systems will need to be considered (to prevent the geometry of the simulation box from biasing the system's evolution); moreover, a refinement of the relevant parameters may be required. Regarding the simulation framework, the ELBA force field is currently implemented only in our in-house software [43], which does not yet offer the flexibility and performance features typical of the most popular molecular dynamics programs. For example, our software is not currently equipped for parallel calculations; this clearly limits the range of potential applications of the model. While efforts are ongoing to improve our code, work has also started to implement ELBA into a mainstream parallel molecular dynamics program [138], [139]; preliminary tests, reported in the Supporting Information S1, are promising.

A potentially controversial issue involves the lack of long-range electrostatics in the ELBA force field. In fact, while we include electrostatic interactions explicitly up to the cutoff distance, the remaining contribution (beyond the cutoff) is neglected. We are aware that this approximation has been shown to introduce simulation artifacts [92]. However, issues and artifacts have also been observed in atomistic simulations where long-range electrostatic interactions are included by standard Ewald techniques [140], [141]. In fact, it has been argued that cutoff approximations can be as good as [142]–[144] or better [141] than Ewald methods. Overall, we believe that for the ELBA model it is appropriate to use the cutoff approximation; in fact, cutoff methods are unquestionably simpler and more computationally efficient than long-range methods, and hence they are arguably more consistent with the overall simplification spirit at the heart of coarse-grain modeling.

In terms of future developments, the force field will be extended to include additional lipid species; in fact, preliminary studies are being conducted to model ceramide, which is a major component of the skin, and also a lipid capable of forming microdomains believed to play crucial roles in signaling processes [60]. The ELBA force field could also be extended to model proteins, possibly providing significant advantages over existing approaches, especially in terms of electrostatics. For example, the protein backbone of most CG models is represented with apolar particles [145]–[147], thus ignoring the large dipole (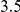 D) that characterizes the peptide bond [148]. In fact, this issue has been recently addressed by the dipole-based CG protein model developed by Alemani et al. [149]; this model could potentially be combined with the ELBA force field. Future prospects will also be focused on multiscale applications. Since our force field includes explicitly the fundamental electrostatics of lipids and water, it is in principle directly compatible with standard atomistic molecular models. In fact, ELBA shares the relevant features with our earlier model [5], [6], which has already been successfully applied to the simulation of multiscale “dual-resolution” systems [16]–[18]. A particularly appealing application will involve the coupling of the ELBA models for lipid and water with standard atomistic models of membrane proteins.

## Supporting Information

Supporting Information S1 Forces and torques for the ELBA potentials, dielectric constant of the ELBA water model, error analysis, additional membrane results, sensitivity to timestep size, benchmarks.
(PDF)
