# Supplementary material for: The ELBA Force Field for Coarse-Grain Modeling of Lipid Membranes
Source: PLoS One. 2011 Dec 16;6(12):e28637. doi: 10.1371/journal.pone.0028637 (PMC3241685; doi:10.1371/journal.pone.0028637)
Supplement: Supporting Information S1 — Forces and torques for the ELBA potentials, dielectric constant of the ELBA water model, error analysis, additional membrane results, sensitivity to timestep size, benchmarks. (PDF) [file pone.0028637.s001.pdf]

# SUPPORTING INFORMATION

**The ELBA Force Field for Coarse-Grain Modeling of Lipid Membranes**

Mario Orsi*, Jonathan W Essex
School of Chemistry, University of Southampton, Southampton, United Kingdom
∗ E-mail: orsi@soton.ac.uk

# Contents

**1 Forces and torques for the ELBA potentials** **1**

**2 Dielectric constant of the ELBA water model** **4**

**3 Error analysis** **6**

**4 Additional membrane results** **7**
4.1 DOPE electron density profiles . . . 7
4.2 DSPC snapshots . . . 8
4.3 Electrostatic potential profiles . . . 9
4.4 Diffusion coefficients . . . 10
4.5 Water permeation . . . 11
4.6 Comparison of self-assembled and pre-assembled systems . . . 12

**5 Sensitivity to timestep size** **13**
5.1 Energy conservation . . . 13
5.2 Membrane properties . . . 14

**6 Benchmarks** **15**

# 1 Forces and torques for the ELBA potentials

The following paragraphs contain the explicit expressions of forces and (where applicable) torques derived from the potentials employed in the ELBA force field. The distance vector between a pair of interacting sites is defined as:

$$\mathbf{r}_{ij} = \mathbf{r}_i - \mathbf{r}_j, \tag{1}$$

with $\mathbf{r}_i$ and $\mathbf{r}_j$ the position vectors of (respectively) site $i$ and site $j$. For an interacting pair $(i, j)$, only the force $\mathbf{f}_{ij}$ will be reported, because $\mathbf{f}_{ij} = -\mathbf{f}_{ji}$ (Newton's third law). The torques $\mathbf{T}_{ij}$ and $\mathbf{T}_{ji}$ are connected through the expression for the local conservation of angular momentum [1]:

$$\mathbf{T}_{ij} + \mathbf{T}_{ji} + \mathbf{r}_{ij} \times \mathbf{f}_{ij} = 0 \tag{2}$$

therefore, both torques $\mathbf{T}_{ij}$ and $\mathbf{T}_{ji}$ will be reported. In general, the various symbols in the expressions reported below are defined in the main body of the paper for each corresponding potential.

### Shifted-force Lennard-Jones potential

$$\mathbf{f}_{ij} = 24\epsilon \left\{ \left[ 2\left(\frac{\sigma}{r_{ij}}\right)^{12} - \left(\frac{\sigma}{r_{ij}}\right)^{6} \right] \frac{1}{r_{ij}^2} - \left[ 2\left(\frac{\sigma}{r_c}\right)^{12} - \left(\frac{\sigma}{r_c}\right)^{6} \right] \frac{1}{r_c^2} \right\} \mathbf{r}_{ij} \tag{3}$$

**Shifted-force charge-charge potential**

$$\mathbf{f}_{ij} = \frac{Q_i Q_j}{4\pi\varepsilon_0 r_{ij}^3}\left(1 - \frac{r_{ij}}{r_c}\right)\left(1 + \frac{r_{ij}}{r_c}\right)\mathbf{r}_{ij}, \tag{4}$$

**Shifted-force charge-dipole potential**

Assuming $i$ is the charge and $j$ is the point-dipole:

$$\mathbf{f}_{ij} = \frac{Q_i \mu_j}{4\pi\varepsilon_0 r_{ij}^3}\left\{3\frac{\boldsymbol{\mu}_j \mathbf{r}_{ij}}{|\boldsymbol{\mu}_j| r_{ij}^2}\left[1 - \left(\frac{r_{ij}}{r_c}\right)^2\right]\mathbf{r}_{ij} - \left[1 - 3\left(\frac{r_{ij}}{r_c}\right)^2 + 2\left(\frac{r_{ij}}{r_c}\right)^3\right]\frac{\boldsymbol{\mu}_j}{|\boldsymbol{\mu}_j|}\right\} \tag{5}$$

$$\mathbf{T}_{ji} = \frac{Q_i \mu_j}{4\pi\varepsilon_0 r_{ij}^3}\left[1 - 3\left(\frac{r_{ij}}{r_c}\right)^2 + 2\left(\frac{r_{ij}}{r_c}\right)^3\right]\left(\mathbf{r}_{ij} \times \frac{\boldsymbol{\mu}_j}{|\boldsymbol{\mu}_j|}\right) \tag{6}$$

Since $i$ is a point-particle, $\mathbf{T}_{ij} = 0$.

**Switched dipole-dipole potential**

Following Allen and Tildesley [2, p. 332], we define:

$$\hat{\mathbf{e}}_i = \frac{\boldsymbol{\mu}_i}{|\boldsymbol{\mu}_i|} \qquad \hat{\mathbf{e}}_j = \frac{\boldsymbol{\mu}_j}{|\boldsymbol{\mu}_j|} \tag{7}$$

$$\cos\gamma_{ij} = \hat{\mathbf{e}}_i\,\hat{\mathbf{e}}_j \qquad \cos\theta_i = \frac{\hat{\mathbf{e}}_i\,\mathbf{r}_{ij}}{r_{ij}} \qquad \cos\theta_j = \frac{\hat{\mathbf{e}}_j\,\mathbf{r}_{ij}}{r_{ij}} \tag{8}$$

For $r_{ij} \leq r_s$:

$$\mathbf{f}_{ij} = \frac{3\mu^2}{r_{ij}^4}\left[(\cos\gamma_{ij} - 5\cos\theta_i\cos\theta_j)\left(\frac{\mathbf{r}_{ij}}{r_{ij}}\right) + \hat{\mathbf{e}}_i\cos\theta_j + \hat{\mathbf{e}}_j\cos\theta_i\right] \tag{9}$$

$$\mathbf{T}_{ij} = -\frac{\mu^2}{r_{ij}^3}\left[\hat{\mathbf{e}}_i \times \hat{\mathbf{e}}_j - 3\cos\theta_j(\hat{\mathbf{e}}_i \times \mathbf{r}_{ij})/r_{ij}\right] \tag{10}$$

$$\mathbf{T}_{ji} = -\frac{\mu^2}{r_{ij}^3}\left[\hat{\mathbf{e}}_j \times \hat{\mathbf{e}}_i - 3\cos\theta_i(\hat{\mathbf{e}}_j \times \mathbf{r}_{ij})/r_{ij}\right] \tag{11}$$

For $r_s < r_{ij} < r_c$:

$$\mathbf{f}_{ij}^s = \mathbf{f}_{ij}s_{ij} - \frac{|\boldsymbol{\mu}_i||\boldsymbol{\mu}_j|}{r_{ij}^4}(\cos\gamma_{ij} - 3\cos\theta_i\cos\theta_j)\frac{6(r_c - r_{ij})(r_s - r_{ij})}{(r_c - r_s)^3}\mathbf{r}_{ij} \tag{12}$$

$$\mathbf{T}_{ij}^s = \mathbf{T}_{ij}s_{ij} \tag{13}$$

$$\mathbf{T}_{ji}^s = \mathbf{T}_{ji}s_{ij} \tag{14}$$

where:

$$s_{ij} = \frac{(r_c + 2r_{ij} - 3r_s)(r_c - r_{ij})^2}{(r_c - r_s)^3} \tag{15}$$

**Hook (harmonic) potential**

$$\mathbf{f}_{ij} = -k(l_{ij} - l_0)\frac{\mathbf{r}_{ji}}{|\mathbf{r}_{ji}|} \tag{16}$$

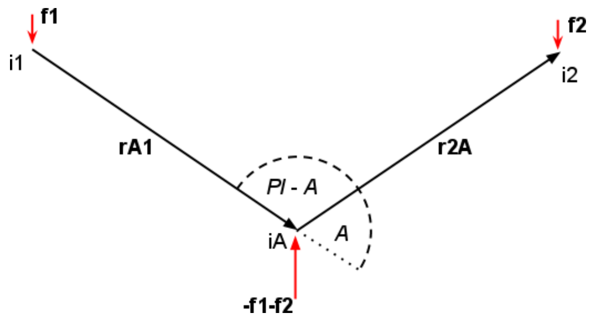


**Figure 1.** Angle-bending.

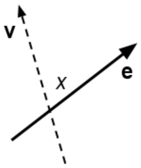


**Figure 2.** Orientation-restraining potential. The "free" vector **e** and the "reference" vector **v** form the angle $x$.

## Angle-bending

The potential $U(A)$ associated with bond angle variation is [3]:

$$U(A) = \frac{w}{2}(\cos A - \cos A_0)^2 \tag{17}$$

with $w$ the rigidity constant, $A$ the actual angle and $A_0$ the reference angle (see Figure 1). Forces and torques can be found in the original reference [3, pp. 283-285].

## Orientation-restraining potential

Consider two unit vectors **v** and **e** forming an angle $x$ between them (Fig. 2). To restrain the "free" vector **e** to lie along the direction defined by the "reference" vector **v** we can use the potential $U$:

$$U = \frac{c}{2}(\cos x - 1)^2 \tag{18}$$

with $c$ a rigidity constant and $\cos x = \mathbf{v} \cdot \mathbf{e}$. This orientation-restraining interaction is required to generate a torque on **e** to favor alignment along **v**. Such a torque $\mathbf{T_e}$ is defined by [2, p. 333]:

$$\mathbf{T_e} = -\mathbf{e} \times \nabla_\mathbf{e} U \tag{19}$$

Applying the chain rule, we can write:

$$\nabla_\mathbf{e} U = \left(\frac{\partial U}{\partial \cos x}\right) \nabla_\mathbf{e} \cos x \tag{20}$$

Solving the derivatives, we obtain:

$$\left(\frac{\partial U}{\partial \cos x}\right) = c(\cos x - 1) \tag{21}$$

and:

$$\nabla_{\mathbf{e}} \cos x = \nabla_{\mathbf{e}} (\mathbf{v} \cdot \mathbf{e}) = \mathbf{v} \tag{22}$$

The final expression is therefore:

$$\mathbf{T}_{\mathbf{e}} = c(1 - \cos x)\, \mathbf{e} \times \mathbf{v} \tag{23}$$

Note that $\mathbf{T}_{\mathbf{e}}$ always induces a rotation that tends to align $\mathbf{e}$ with the reference vector $\mathbf{v}$. Also note that there is no force arising from the potential of equation 18. In fact, the interaction force is in general obtained from the gradient of the potential with respect to the vector $\mathbf{r}$ representing the distance from the origin. Since in this case the potential does not depend on such a vector, the interaction force is zero ($\mathbf{f} = -\nabla_{\mathbf{r}} U = 0$).

# 2 Dielectric constant of the ELBA water model

The ELBA force field does not include long-range interactions. Since these are necessary to calculate the dielectric constant, for this purpose we modified the standard ELBA potential $U$ by the addition of a reaction field potential $U_{ij}^{RF}$, so that the new total potential energy $U^*$ becomes:

$$U^* = U + U_{ij}^{RF} \tag{24}$$

where $U$ includes the various components described in the main body of the paper.

**Switched reaction field potential** We consider a standard dipole-dipole reaction field potential [4,5] supplemented by a cubic switching function $s_{ij}$:

$$U_{ij}^{RF} = -\frac{\boldsymbol{\mu}_i \cdot \boldsymbol{\mu}_j}{4\pi\varepsilon_0 r_\alpha^3} s_{ij}, \text{ with } s_{ij} = \begin{cases} 1 & \text{if } r_{ij} \le r_s \\ \frac{(r_c - r_{ij})^2 (r_c + 2r_{ij} - 3r_s)}{(r_c - r_s)^3} & \text{if } r_s < r_{ij} < r_c \end{cases} \tag{25}$$

where $r_\alpha^3$ is a modified radius which takes into account the effects of a switching function, and the other symbols have the same meaning as in the equations for the dipole-dipole potential reported previously. In particular, $r_\alpha^3$ is defined as [6]:

$$r_\alpha^3 = 3 \int_0^\infty r_{ij}^2\, s_{ij}\, \mathrm{d}r \tag{26}$$

Considering our choice of switching function, equation 26 becomes:

$$r_\alpha^3 = 3 \left[ \int_0^{r_s} r_{ij}^2\, \mathrm{d}r + \int_{r_s}^{r_c} r_{ij}^2 \frac{(r_c - r_{ij})^2 (r_c + 2r_{ij} - 3r_s)}{(r_c - r_s)^3} \mathrm{d}r \right] = 3 \left( \frac{r_c^3}{15} + \frac{r_c^2\, r_s}{10} + \frac{r_c\, r_s^2}{10} + \frac{r_s^3}{15} \right) \tag{27}$$

The dielectric constant of the of the continuum outside $r_c$ is set to infinity, that is, $\epsilon_{RF} = \infty$. The pair force is:

$$\mathbf{f}_{ij} = \begin{cases} 0 & \text{if } r_{ij} \le r_s \\ \frac{\mu^2 \cos\gamma}{4\pi\varepsilon_0 r_\alpha^3} \frac{6(r_c - r_{ij})(r_s - r_{ij})}{r_{ij}(r_c - r_s)^3}\, \mathbf{r}_{ij} & \text{if } r_s < r_{ij} < r_c \end{cases} \tag{28}$$

The pair torques are:

$$\mathbf{T}_{ij} = -\mathbf{T}_{ji} = \begin{cases} \frac{\mu^2}{4\pi\varepsilon_0 r_\alpha^3}\, \hat{\mathbf{e}}_i \times \hat{\mathbf{e}}_j & \text{if } r_{ij} \le r_s \\ \frac{\mu^2 (r_c - r_{ij})^2 (r_c + 2r_{ij} - 3r_s)}{4\pi\varepsilon_0 r_\alpha^3 (r_c - r_s)^3}\, \hat{\mathbf{e}}_i \times \hat{\mathbf{e}}_j & \text{if } r_s < r_{ij} < r_c \end{cases} \tag{29}$$

### Calculation methodology

The calculation of the dielectric constant was carried out using two standard methods.

In particular, using the “fluctuation method”, the dielectric constant can be obtained as [7]:

$$\epsilon_r = 1 + \frac{4\pi}{3}\frac{\langle \mathbf{M}^2 \rangle}{V k_B T} \tag{30}$$

where $\mathbf{M}$ is the total dipole moment of the system, $V$ is the volume of the simulation region, and $T$ is the temperature. The total dipole moment of the system is computed with:

$$\mathbf{M} = \sum_{i=1}^{N} \boldsymbol{\mu}_i \tag{31}$$

where $\boldsymbol{\mu}_i$ is the dipole moment of site $i$.

Using the polarization method, the dielectric constant can be obtained as [8]:

$$\epsilon_r = 1 + 4\pi \frac{\langle P_z \rangle}{E_z^{ext}} \tag{32}$$

where $P_z$ is the system dipole moment along the direction of the applied field $E_z^{ext}$. We recall that the potential energy of a point-dipole $\boldsymbol{\mu}$ in an external electric field $\mathbf{E}$ is:

$$U = -\boldsymbol{\mu} \cdot \mathbf{E} = -\mu E \cos\theta \tag{33}$$

with $\theta$ the angle between the two vectors. This generates a torque on the point-dipole:

$$\mathbf{T}_\mu = \boldsymbol{\mu} \times \mathbf{E} \tag{34}$$

## Results

To estimate the dielectric constant of the ELBA water model, we carried out simulations of water systems comprising 4000 sites. Unfortunately, we found that the addition of the RF potential (equation 25) to the ELBA model causes an unphysical phenomenon; in particular, the molecular dipoles align along a preferential orientation, incidentally causing the dielectric constant calculation to break down. Such artificial ordering effects could be removed by reducing the switching radius $r_s$ of the RF potential; the results obtained are collected in Table 1.

**Table 1. Dielectric constant calculation for the ELBA water model**

| RF switching radius | $0.6\,r_c$ | | $0.7\,r_c$ | | $0.8\,r_c$ | |
|---|---|---|---|---|---|---|
| Calculation method | Fluct | Pol | Fluct | Pol | Fluct | Pol |
| $T = 30°$C | $28.2 \pm 0.2$ | $26.8 \pm 0.2$ | $66.7 \pm 0.7$ | $71.3 \pm 0.6$ | $221.6 \pm 1.4$ | $166.0 \pm 0.2$ |
| $T = 25°$C | $29.5 \pm 3.7$ | $26.7 \pm 0.1$ | $70.7 \pm 0.9$ | $72.4 \pm 0.2$ | $205.4 \pm 2.2$ | $169.0 \pm 0.3$ |

The dielectric constant calculations are carried out with both the fluctuation (“Fluct”) and the polarization (“Pol”) methods. For the polarization method, the external electric field $E_z^{ext}$ (see equation 32) was set to 0.01 V.

It is clear that the results are highly sensitive to the cutoff treatment of the reaction field potential; such a finding was not completely unexpected, and in fact a similar phenomenon has been observed previously in the literature [9–11]. In particular, it can be seen from Table 1 that increasing values of the RF switching radius (from $0.6\,r_c$ to $0.8\,r_c$ ) result in increasing values of the dielectric constant. For switching radii

set to $0.9\,r_c$ and $1.0\,r_c$ (corresponding to straight cutoff), the calculation of the dielectric constant breaks down, in that the reaction field causes the water dipole to align along a preferential direction. In general, any truncation scheme effectively changes the potential, and hence it is bound to alter the physical properties of the model. The ELBA model seems to be especially sensitive to the additional reaction field potential; while this behavior is not ideal, it only affects the calculation of the dielectric constant. For all other purposes, the ELBA force field does not require the addition of a reaction field.

An alternative method to estimate the dielectric constant would involve treating the long-range dipolar interactions through a lattice summation technique [7]. While outside the scope of this paper, future work will be devoted to this issue; this will require the implementation of a dipole-dipole lattice summation scheme in our software.

# 3 Error analysis

The numerical properties reported in the "Results" section of the main body of the paper are reported in the form *average* ± *standard error*, where the average was calculated from the entire simulation, and the standard errors were obtained by dividing each trajectory into two consecutive blocks. Considering a property $A$, evaluated in a number $M$ of measurements, the average $\langle A \rangle$ was obtained as:

$$\langle A \rangle = \frac{1}{M} \sum_{\mu=1}^{M} A_\mu \tag{35}$$

where $A_\mu$ represents a single measurement. The following properties were evaluated at every step: $A_\mathrm{L}$, $V_\mathrm{L}$, $\mu_\mathrm{HG}$, $\theta_\mathrm{HG}$. In this case, the number of measurements $M$ is obviously the same as the number of simulation steps. The following properties, and associated parameters, were instead evaluated every 100 steps (corresponding to 1.5 ps using a 15 fs timestep): electron density, lateral pressure, electrostatic potential. In this case, $M$ = total number of steps/100. For every property, two subaverages $A_1$ and $A_2$ were calculated over the two consecutive halves of the trajectory of each simulation. The standard deviation $s_d$ was then computed as:

$$s_d = \sqrt{\frac{1}{N-1} \sum_{i=1}^{N} (A_i - \langle A \rangle)^2} = \sqrt{[(A_1 - \langle A \rangle)^2 + (A_2 - \langle A \rangle)^2]} \tag{36}$$

where $N$ is the number of subaverages. The standard error $s_e$ was eventually computed with:

$$s_e = \frac{s_d}{\sqrt{N}} = \frac{s_d}{\sqrt{2}} \tag{37}$$

We are aware that, for the error estimation to be reliable, the subaverages must be statistically independent; this can be proved rigorously using a block averaging (BA) procedure, as described by Flyvbjierg and Petersen [12]. The BA method should in principle identify the longest correlation time present in the data, thus providing us with a reasonable value for the minimum block length that guarantees statistical independence of the block averages. Unfortunately, while this procedure is relatively straightforward for simple systems where only a few quantities of interest are calculated, it becomes problematic in cases like ours. The various membrane properties calculated are expected to be characterized by different correlation times, also in relation to the specific system size, temperature and presumably hydration; hence the optimal block length would have to be estimated independently for each property in each simulation. In fact, the BA method is not strictly guaranteed to work for large and heterogeneous systems such as lipid bilayers, as the possible existence of very long correlation times might prevent the method from converging over typical simulation timescales. In general, for all these reasons, we should note that BA is hardly ever applied in its rigorous form to the analysis of molecular dynamics data from complex biomolecular simulations. Overall, we believe that our sampling times are long enough to provide reliable averages and error estimations, as they are 1-2 orders of magnitude larger than what is normally achieved by standard atomistic models.

# 4 Additional membrane results

## 4.1 DOPE electron density profiles

The electron density profiles calculated from our simulations of DOPE bilayers are reported in Figure 3.

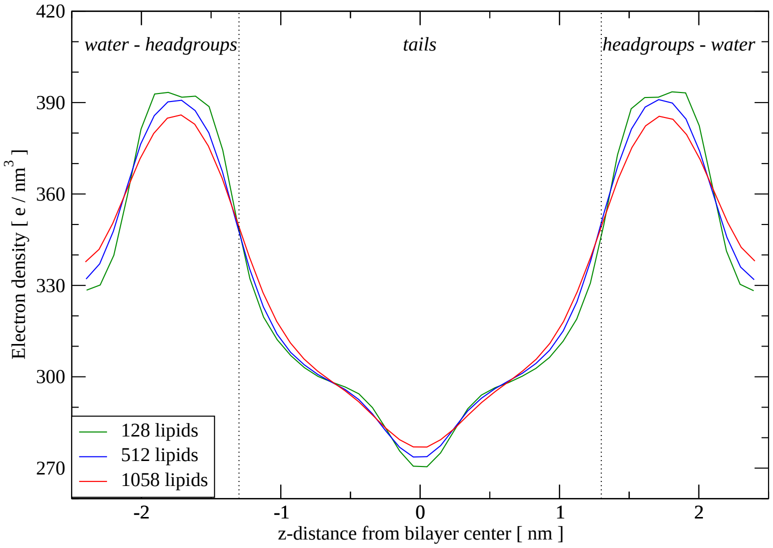


**Figure 3. DOPE electron density profiles.** The curves refer to runs D (128 lipids), E (512 lipids) and F (1058 lipids).

A slight size dependence can be noticed; the origin of this artifact is discussed in the main body of the paper.

## 4.2 DSPC snapshots

Figure 4 presents a snapshot from a simulation of a DSPC bilayer at 60°C; this image can be compared with the snapshots from gel-phase simulation reported in the main body of the paper.

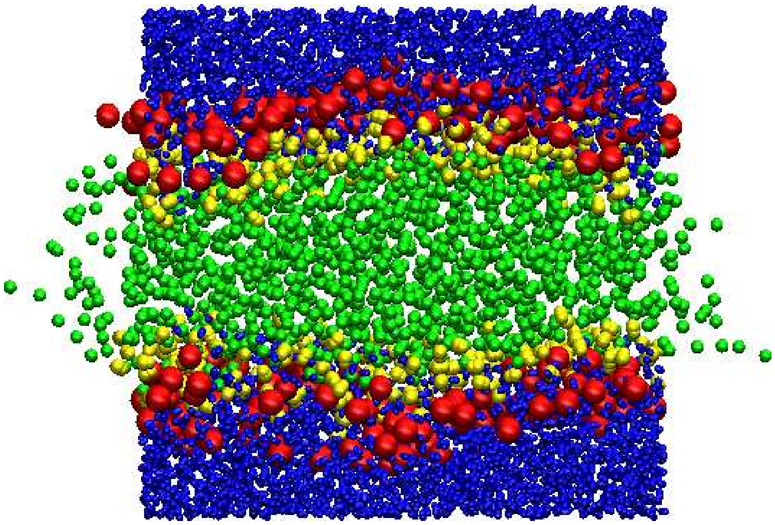

**Figure 4. Liquid-phase DSPC bilayer.** Snapshot from a simulation of 128 DSPC lipids and 4232 water molecules at 60°C.

A qualitative comparison between typical conformations of single lipids in the fluid and gel phase is displayed in Figure 5.

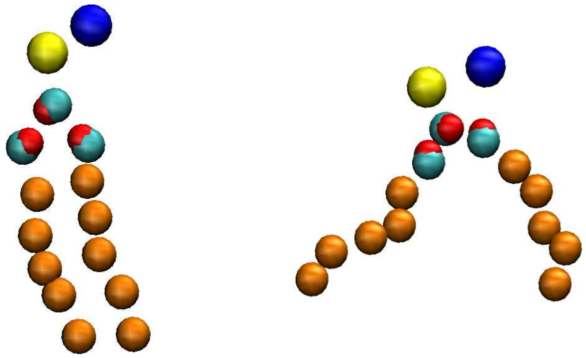

**Figure 5. Lipid conformations.** Snapshots of single lipids from simulation of DSPC bilayers in the gel-phase (left) and in the fluid-phase (right).

The gel-phase conformation is characterized by parallel and “stretched out” tails, in contrast with the “disordered” fluid-phase conformation.

## 4.3 Electrostatic potential profiles

The electrostatic potential profiles calculated from our simulations of DOPC, DOPE and DSPC are reported respectively in Figure 6, 7 and 8.

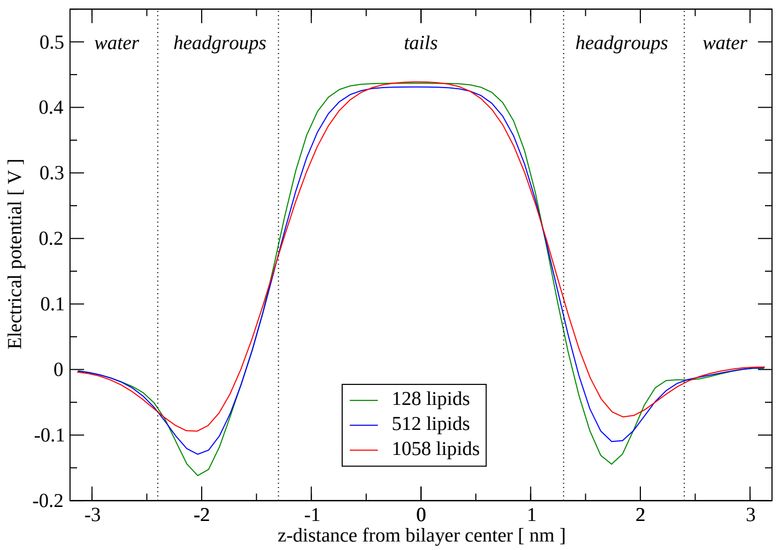


**Figure 6. DOPC electrostatic potential profiles.** The curves refer to runs A (128 lipids), B (512 lipids) and C (1058 lipids).

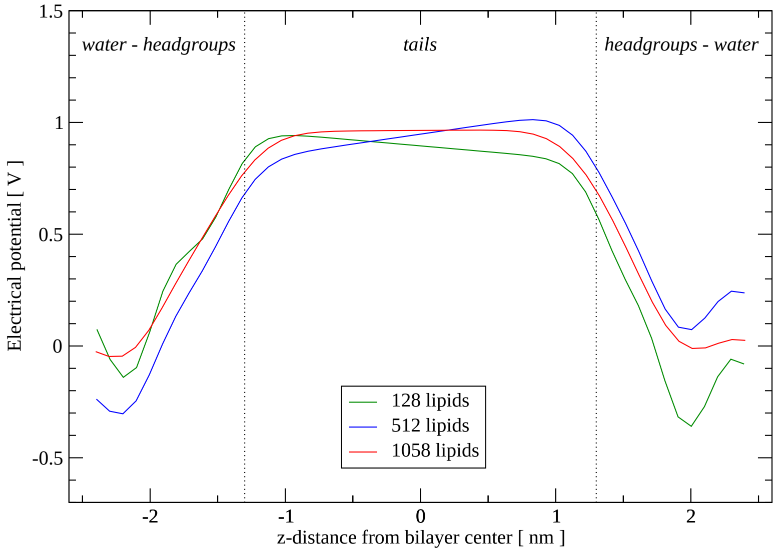


**Figure 7. DOPE electrostatic potential profiles.** The curves refer to runs D (128 lipids), E (512 lipids) and F (1058 lipids).

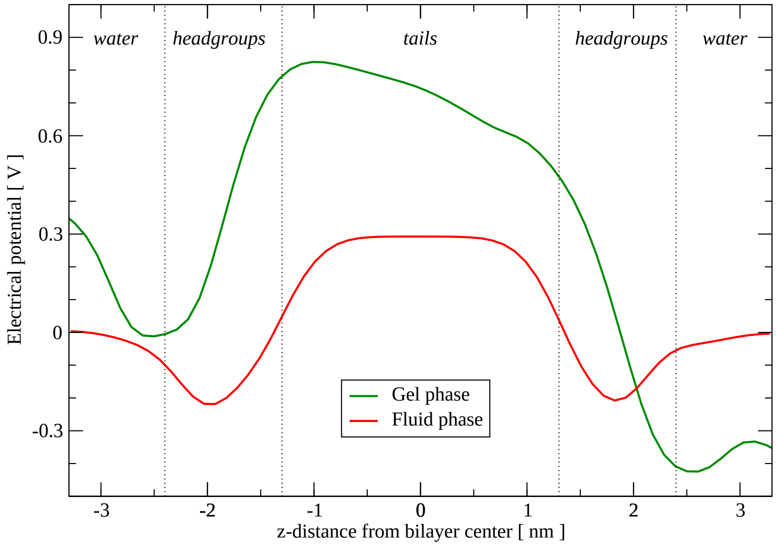


**Figure 8. DSPC electrostatic potential profiles.** The "gel phase" curve refers to the simulation at 30°C (run G), while the "fluid phase" curve refers to the simulation at 60°C (run H).

## 4.4 Diffusion coefficients

The diffusion coefficients as a function of the measurement time for runs A, D G and H are reported in Figure 9.

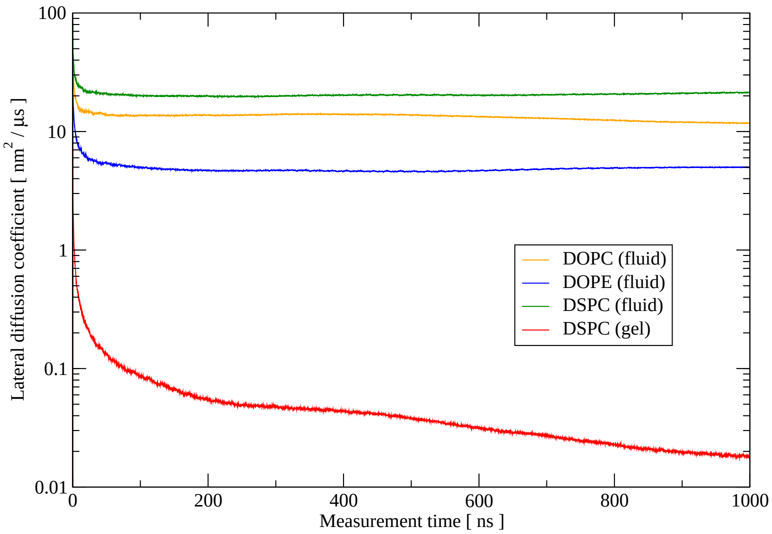


**Figure 9. Lateral diffusion coefficients.** Lateral diffusion coefficients of lipid mass-centers as a function of the measurement time.

It can be seen that the lateral diffusion coefficient for gel DSPC is $2-3$ orders of magnitude lower than those obtained for fluid bilayers.

### 4.5 Water permeation

Table 2 collects the ratios between water permeability coefficients through PC and PE bilayers from our calculations and from the measurements reported in the literature.

**Table 2. Ratios of permeability coefficients through PC and PE membranes**

| | PC species | PE species | Ratio $P_{PC}/P_{PE}$ |
|---|---|---|---|
| This work | 18:1-18:1 PC (DOPC) | 18:1-18:1 PE (DOPE) | 2.9 (runs A,D), 2.8 (runs B,E), 3.6 (runs C,F) |
| Exp [13] | 16:1-16:1 PC (DPPC) | 16:1-16:1 PE (DPPE) | 1.3 ($T = 20°$C), 81 ($T = 70°$C) |
| Exp [13] | 14:1-14:1 PC (DMPC) | 14:1-14:1 PE (DMPE) | 0.56 ($T = 20°$C), 226 ($T = 70°$C) |
| Exp [14] | 12:1-12:1 PC (DLPC) | 12:1-12:1 PE (DLPE) | 4.9 ($T = 30°$C) |

Table 3 collects the ratios between water permeability coefficients through gel and fluid DSPC bilayers from our calculations and from experimental measurements reported in the literature.

**Table 3. Ratios of permeability coefficients through gel and fluid DSPC membranes**

| Method [reference] | Gel temperature | Fluid temperature | Ratio $P_{gel}/P_{fluid}$ |
|---|---|---|---|
| Simulation [this work] | 30°C | 60°C | 0.005 |
| Diffusive [13] | 20°C | 70°C | 0.002 |
| Osmotic [13] | 20°C | 70°C | 0.003 |

### 4.6 Comparison of self-assembled and pre-assembled systems

Figures 10, 11 and 12 show comparative diagrams for the results obtained from analysis of a self-assembled DOPC bilayers and a corresponding pre-assembled system. It is clear that both sets of calculations yield substantially equivalent results.

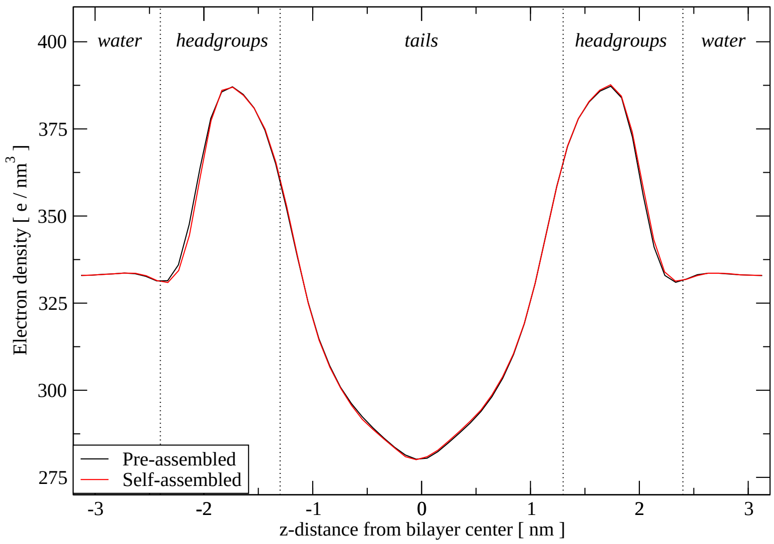


**Figure 10. DOPC electron density profiles.** Comparison between electron density profiles obtained from self-assembled and pre-assembled systems.

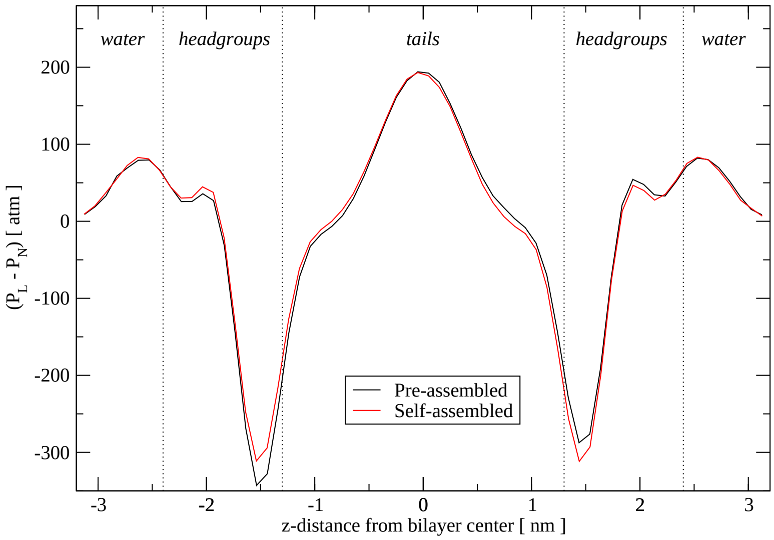


**Figure 11. DOPC lateral pressure profiles.** Comparison between lateral pressure profiles obtained from self-assembled and pre-assembled systems.

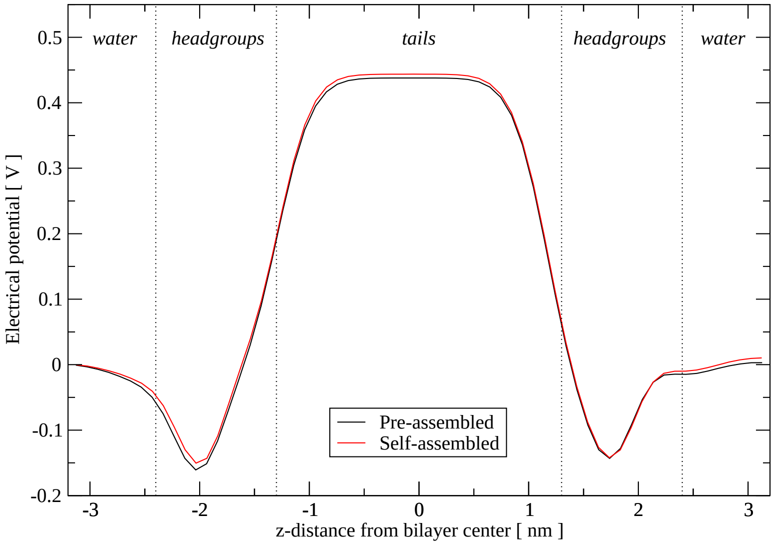


**Figure 12. DOPC electrostatic potential profiles.** Comparison between electrostatic potential profiles obtained from self-assembled and pre-assembled systems.

# 5 Sensitivity to timestep size

## 5.1 Energy conservation

The total and potential energies over 1000 steps for different timestep sizes are plotted in Figure 13.

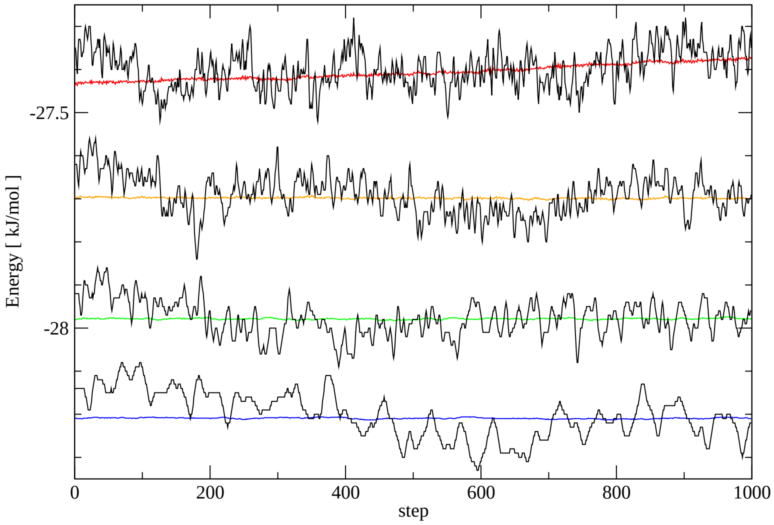


**Figure 13. Energy fluctuations.** Comparison between energy fluctuations for different timestep sizes. Total energies are plotted in color for the following timesteps: 5 fs (blue), 10 fs (green), 15 fs (orange) and 20 fs (red). Potential energies are plotted in black and superimposed to the total energy of the corresponding timestep size. Curves have been shifted along the $y$ axis for clarity.

It can be seen that the energy is conserved up to a timestep size of 15 fs.

## 5.2 Membrane properties

Figures 14, 15, 16 and 17 show comparative diagrams for the results obtained from four 150-ns long simulations of a DOPC bilayers simulated with four different integration timesteps. It can be seen that the results obtained are consistent with each other.

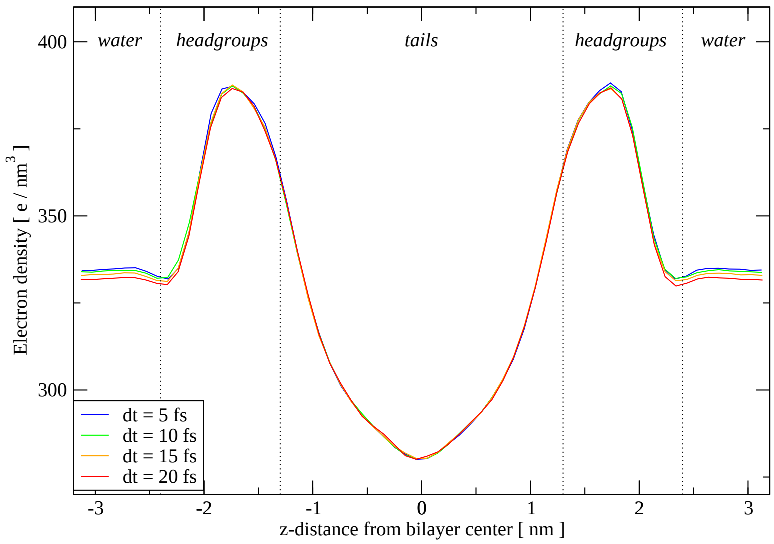


**Figure 14. DOPC electron density profiles.** Comparison between electron density profiles obtained from self-assembled and pre-assembled systems.

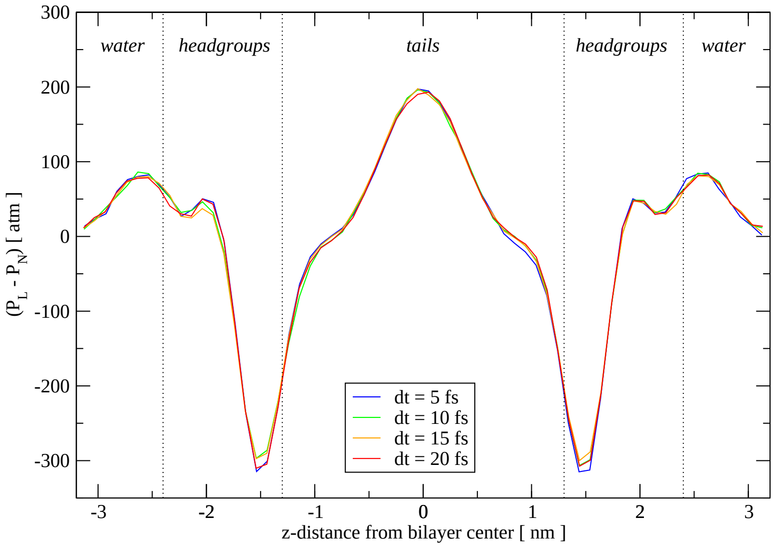


**Figure 15. DOPC lateral pressure profiles.** Comparison between lateral pressure profiles obtained from self-assembled and pre-assembled systems.

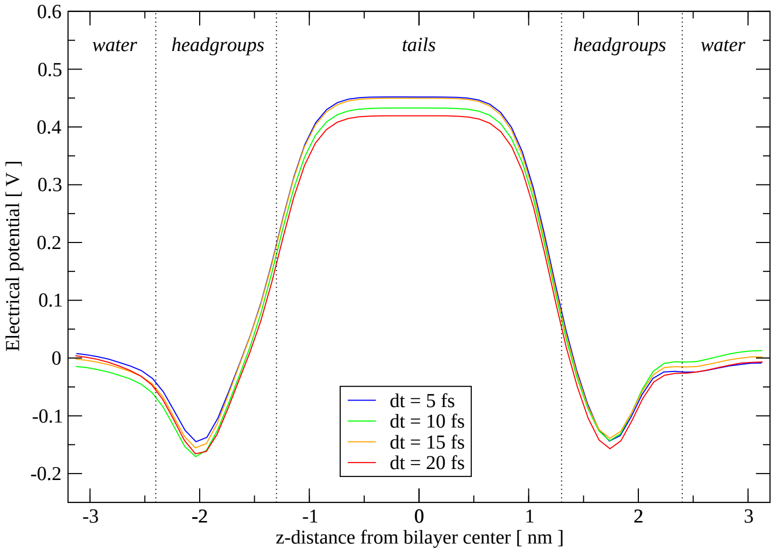


**Figure 16. DOPC electrostatic potential profiles.** Comparison between electrostatic potential profiles obtained from self-assembled and pre-assembled systems.

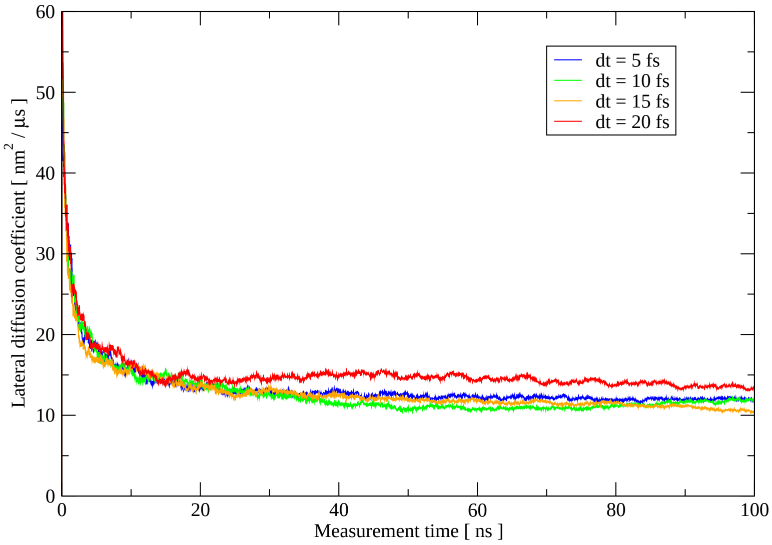


**Figure 17. Lateral diffusion coefficients.** Lateral diffusion coefficients of lipid mass-centers as a function of the measurement time.

# 6 Benchmarks

This section presents an assessment of the computational efficiency of the ELBA coarse-grain model. The model was run with the serial program Brahms [15] and with the parallel program Lammps [16]. The benchmark system employed comprises 1152 lipids and 17328 water sites. To compare the ELBA performances with standard atomistic models, we prepared two roughly equivalent systems using a united-atom (UA) and an all-atom (AA) force field [17, 18]. These atomistic systems were run with the popular

Gromacs software [19]. The various simulation parameters (timestep, cutoff radii, long-range schemes) were set following standard protocols [18]. All data reported in this section were obtained on the Iridis supercomputer [20] using identical processors (2.27 Ghz Nehalem).

For each system, we quantified the efficiency by calculating the "intrinsic speed" of the molecular dynamics simulation as:

$$\text{MD speed} = \frac{\text{\# molecular dynamics steps}}{\text{second}} \tag{38}$$

where "second" is intended in terms of calculation (wall-clock) time. Moreover, we calculated the overall sampling efficiency as:

$$\text{Sampling speed} = \frac{\text{\# nanoseconds sampled}}{\text{day}} \tag{39}$$

where "day" is intended in terms of calculation (wall-clock) time.
The results obtained for single-processor simulations are collected in table 4.

**Table 4. Comparison of performances on a single processor (2.27 Ghz Nehalem)**

| Identifier | ELBA-Brahms | ELBA-Lammps | UA-Gromacs | AA-Gromacs |
|---|---|---|---|---|
| Force field | ELBA [this work] | ELBA [this work] | United-Atom [17] | All-Atom [18] |
| # lipids | 1152 (DOPC) | 1152 (DOPC) | 1152 (POPC) | 1152 (POPC) |
| # waters | 17328 (ELBA) | 17328 (ELBA) | 17541 (SPC) | 17577 (TIP) |
| # equivalent atoms | 210960 | 210960 | 207099 | 207099 |
| # simulated sites | 34608 | 34608 | 112527 | 207099 |
| Software | Brahms [15] | Lammps [16] | Gromacs 4.5.1 [19] | Gromacs 4.5.1 [19] |
| Cutoff radii [Å] | 9 (water), 12 (rest) | 12 (all) | 9 (Coul), 14 (LJ) | 12 (Coul), 12 (LJ) |
| Long-range forces | No | No | PME [21] | PME [21] |
| MD speed [steps/s] | 4.20 | 3.25 | 1.59 | 0.31 |
| Timestep [fs] | 15 | 15 | 2 | 1 |
| Sampling speed [ns/day] | 5.44 | 4.21 | 0.254 | 0.027 |

Further tests were carried out to investigate the "parallel performances" of the same calculations when run on multiple processors using standard domain-decomposition message-passing methods (this was not done for the "ELBA-Brahms" simulation because Brahms does not run in parallel). These tests were run on the Iridis supercomputer [20], which is equipped with 8-processor nodes (each processor being a 2.27 Ghz Nehalem). Figure 18 reports the parallel efficiency as a function of the number of processors used (where "parallel efficiency" is the ratio of ideal to actual run time[1]).
Figure 19 reports a comparison in terms of sampling speed (as defined in equation 39).

[1] For example, if ideal speed-up corresponds to a run-time of 10 seconds, and the actual run time was 12 seconds, then the efficiency is 10/12 or 83.3%.

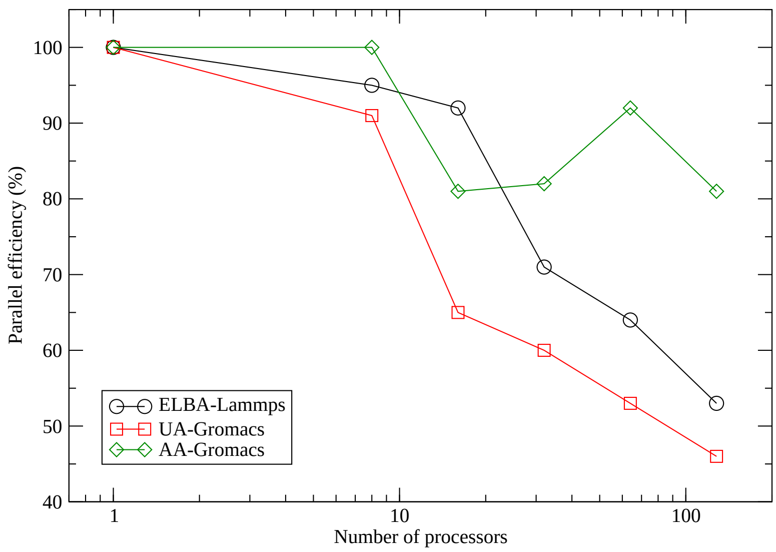


**Figure 18. Parallel efficiency.** Comparison of parallel performances on 1, 8, 16, 32, 64 and 128 processors.

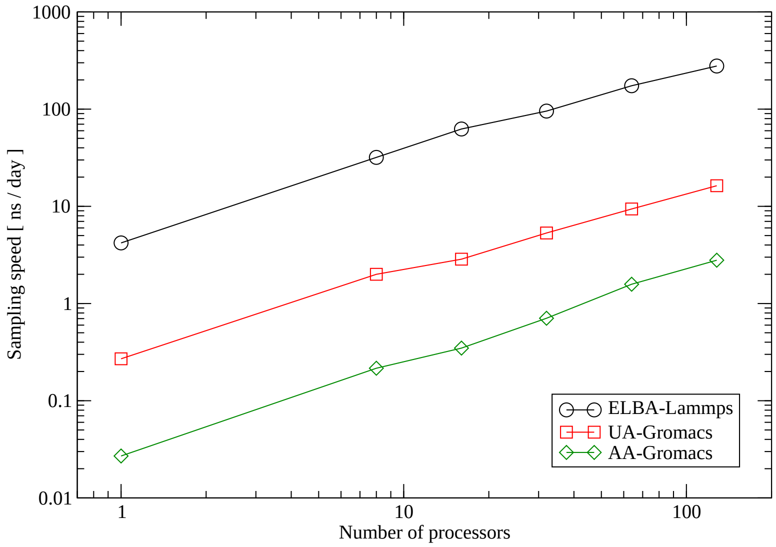


**Figure 19. Sampling speed.** Comparison of parallel performances on 1, 8, 16, 32, 64 and 128 processors.

# References


1. Allen MP, Germano G (2006) Expressions for forces and torques in molecular simulations using rigid bodies. Mol Phys 104: 3225-3235.

2. Allen MP, Tildesley DJ (1987) Computer Simulation of Liquids. Oxford: Oxford Science Publications, 1st edition.

3. Rapaport DC (2004) The art of molecular dynamics simulation. Cambridge: Cambridge University Press, 2nd edition.

4. Liu Y, Ichiye T (1996) The static dielectric constant of the soft sticky dipole model of liquid water: Monte Carlo simulation. Chem Phys Lett 256: 334-340.

5. Johnson LE, Barnes R, Draxler TW, Eichinger BE, Robinson BH (2010) Dielectric constants of simple liquids: Stockmayer and ellipsoidal fluids. J Phys Chem B 114: 8431-8440.

6. Adams DJ, Adams EM, Hills GJ (1979) The computer simulation of polar liquids. Mol Phys 38: 387-400.

7. Neumann M (1983) Dipole moment fluctuation formulas in computer simulations of polar systems. Mol Phys 50: 841-858.

8. Riniker S, van Gunsteren WF (2011) A simple, efficient polarizable coarse-grained water model for molecular dynamics simulations. J Chem Phys 134: 084110.

9. Alper HE, Levy RM (1989) Computer simulations of the dielectric properties of water: Studies of the simple point charge and transferrable intermolecular potential models. J Chem Phys 91: 1242-1251.

10. Essex JW (1998) The application of the reaction-field method to the calculation of dielectric constants. Mol Simulat 20: 159-178.

11. van der Spoel D, van Maaren PJ, Berendsen HJC (1998) A systematic study of water models for molecular simulation: Derivation of water models optimized for use with a reaction field. J Chem Phys 108: 10220-10230.

12. Flyvbjerg H, Petersen HG (1989) Error estimates on averages of correlated data. J Chem Phys 91: 461-466.

13. Jansen M, Blume A (1995) A comparative study of diffusive and osmotic water permeation across bilayers composed of phospholipids with different head groups and fatty acyl chains. Biophys J 68: 997-1008.

14. Mathai JC, Tristram-Nagle S, Nagle JF, Zeidel ML (2008) Structural determinants of water permeability through the lipid membrane. J Gen Physiol 131: 69-76.

15. BRAHMS software website, `http://www.soton.ac.uk/~orsi/brahms`. Accessed 2011 Nov 15.

16. LAMMPS software website, `http://lammps.sandia.gov`. Accessed 2011 Nov 15.

17. Berger O, Edholm O, Jähnig F (1997) Molecular dynamics simulations of a fluid bilayer of dipalmitoylphosphatidylcholine at full hydration, constant pressure, and constant temperature. Biophys J 72: 2002-2013.

18. Klauda JB, Venable RM, Freites JA, O'Connor JW, Tobias DJ, et al. (2010) Update of the CHARMM all-atom additive force field for lipids: Validation on six lipid types. J Phys Chem B 114: 7830-7843.

19. Hess B, Kutzner C, van der Spoel D, Lindahl E (2008) Gromacs 4: Algorithms for highly efficient, load-balanced, and scalable molecular simulation. J Chem Theory Comput 4: 435-447.

20. Iridis supercomputer website, `http://cmg.soton.ac.uk/iridis`. Accessed 2011 Nov 15.

21. Darden T, York D, Pedersen L (1993) Particle mesh ewald - An Nlog(N) method for Ewald sums in large systems. J Chem Phys 98: 10089-10092.
